# ScGAI is a key regulator of culm development in sugarcane

**DOI:** 10.1093/jxb/ery180

**Published:** 2018-05-21

**Authors:** Rafael Garcia Tavares, Prakash Lakshmanan, Edgar Peiter, Anthony O’Connell, Camila Caldana, Renato Vicentini, José Sérgio Soares, Marcelo Menossi

**Affiliations:** 1 Functional Genome Laboratory, Department of Genetics, Evolution and Bioagents, Institute of Biology, State University of Campinas, Campinas, Brazil; 2 Sugar Research Australia (SRA), Indooroopilly, Brisbane, Australia; 3 Plant Nutrition Laboratory, Institute of Agricultural and Nutritional Sciences, Faculty of Natural Sciences III, Martin Luther University Halle-Wittenberg, Halle (Saale), Germany; 4 Brazilian Bioethanol Science and Technology Laboratory, Brazilian Center for Research in Energy and Materials (CTBE), Campinas, Brazil; 5 Max Planck Partner Group at CTBE, Campinas, Brazil; 6 System Biology Laboratory, Department of Genetics, Evolution and Bioagents, Institute of Biology, State University of Campinas, Campinas, Brazil

**Keywords:** Culm development, DELLA, gibberellin, ScGAI, source–sink, sugarcane, SUMOylation

## Abstract

Sugarcane contributes more than 70% of sugar production and is the second largest feedstock for ethanol production globally. Since sugar accumulates in sugarcane culms, culm biomass and sucrose content are the most commercially important traits. Despite extensive breeding, progress in both cane yield and sugar content remains very slow in most countries. We hypothesize that manipulating the genetic elements controlling culm growth will alter source–sink regulation and help break down the yield barriers. In this study, we investigate the role of sugarcane *ScGAI*, an ortholog of *SLR1*/*D8*/*RHT1*/*GAI*, on culm development and source–sink regulation through a combination of molecular techniques and transgenic strategies. We show that ScGAI is a key molecular regulator of culm growth and development. Changing *ScGAI* activity created substantial culm growth and carbon allocation changes for structural molecules and storage. ScGAI regulates spatio-temporal growth of sugarcane culm and leaf by interacting with ScPIF3/PIF4 and ethylene signaling elements ScEIN3/ScEIL1, and its action appears to be regulated by SUMOylation in leaf but not in the culm. Collectively, the remarkable culm growth variation observed suggests that ScGAI could be used as an effective molecular breeding target for breaking the slow yield gain in sugarcane.

## Teaser

Modulation of gibberellic acid signaling through ScGAI regulates culm development including change in phytomer production and source–sink regulation in sugarcane.

## Introduction

Sugarcane (*Saccharum* ssp. hybrids) is one of the largest broad-acre crops in the world, producing more than 70% of sugar consumed globally (http://faostat.fao.org/). Grown in 106 countries spread across the tropics and subtropics, it is the second largest feedstock for biofuel production worldwide (http://faostat.fao.org/). Sugarcane-based commercial bioethanol production is an integral component of sugar industries in many countries, with the Brazilian program Proálcool being expected to produce 65 billion liters of ethanol for liquid fuel use by 2020 (Matsuoka *et al.*, 2009). Besides being a major source of sugar and biofuel, sugarcane is also used for producing electricity, organic fertilizers, fodder and several other byproducts (Wei and Li, 2006).

Sugarcane is a large C_4_ graminaceous crop. Commercially grown sugarcane varieties are interspecific hybrids of the domesticated sugar-producing species *Saccharum officinarum* (female) and the wild cane *S. spontaneum* (male) with multiple backcrosses to *S. officinarum* or to commercial-type hybrids. As with other large C_4_ grasses, such as *Miscanthus* and *Erianthus* species, sugarcane is a high biomass crop with commercial cane yield reaching more than 200 tons ha^−1^ year^−1^ in some fully irrigated production areas. More importantly, sugarcane is unique in that it accumulates unusually high levels of sucrose in its culm, reaching up to 668 mM (Welbaum and Meinzer, 1990), making it a very attractive dual-purpose sugar and fuel crop. Further, vegetative propagation, rapid growth, ability to continue multiple crop cycles before replanting, and a relatively low production cost make sugarcane the crop of choice for second-generation biofuel production.

Commercial sugarcane cultivation, however, is constrained by several biotic and abiotic stresses and a relatively long crop cycle, ranging from 10 to 22 months depending on the production region (Tew and Cobill, 2008). More importantly, sugarcane yield, especially sugar yield, in most sugarcane-growing countries has been more or less static for decades (http://faostat.fao.org/). This remarkably slow yield improvement remains the biggest challenge to variety development. Its occurrence, notwithstanding extensive international breeding efforts, advanced agronomy, and effective pest/disease management, suggests a strong developmental control underpinning a yield ceiling. This physical limitation on sugar storage capacity, i.e. the limitation on culm volume, appears to be a significant developmental constraint for cane and sugar yield improvement, and it may, at least in part, explain the slow rate of yield gain persisting in sugar industries worldwide. Breaking this developmental limitation by conventional breeding in any substantial way is proving hard, and little is known about the molecular controls of sugarcane culm development to formulate biotechnological solutions.

Hormones are the key regulators of plant growth and they play a central role in integrating external and internal cues that modulate development (Depuydt and Hardtke, 2011). On this basis, we hypothesize that sugarcane culm development, source–sink relationship, and consequently the yield ceiling in sugarcane is under hormonal regulation, and that the developmental limitation on sink capacity (culm growth) can be modified by manipulating hormonal activity.

Gibberellins are plant growth hormones that are involved in diverse aspects of growth and development. They are extensively exploited for improving growth and yield in many horticultural and agricultural crops (Hedden and Sponsel, 2015). In the recent past, cloning and characterization of GA-signaling components have greatly advanced our understanding of GA action and its regulation. Briefly, bioactive GA is recognized by its receptor GA-INSENSITIVE DWARF1 (GID1) and the GA–GID1 complex binds to the N-terminus of DELLA proteins, repressors of GA action. This triggers the recruitment of the components of the ubiquitin machinery, leading to DELLA degradation and growth promotion (Achard and Genschik, 2009). Thus, GA-induced growth responses are triggered by the rapid degradation of DELLA proteins. Growing evidence suggests DELLA is a central hub for the integration of other hormones and environmental cues to regulate growth and development (Achard *et al.*, 2006; Weiss and Ori, 2007). We therefore studied the growth regulatory role of DELLA in sugarcane, with emphasis on culm development. We hypothesized that modulation of DELLA expression will be an effective way of creating variation in culm growth and chemical composition of biomass in sugarcane.

In sugarcane, sugar and structural carbohydrate contents are strongly negatively co-related, with two-thirds of fixed carbon being used for the synthesis of complex carbohydrates such as cellulose and hemicellulose. Structural carbohydrate content and composition are important considerations for sugarcane as an energy crop. GA is known to regulate cellulose synthesis (Huang *et al.*, 2015) and modification of GA action via DELLA activity is likely to cause variations in structural carbohydrate that favour energy production.

In this study, we altered the expression of sugarcane ScGAI, the growth repressor DELLA, to gain more insight into the molecular basis of culm development in sugarcane. Modulation of GA signaling through *ScGAI* created substantial variation in culm development, by changing phytomer (defined as a unit comprising a node and internode, its axillary buds, and an attached leaf) production and composition in sugarcane. Interestingly, an organ-specific regulation of ScGAI activity was found in this study with ScGAI SUMOylation occurring in a spatio-temporal manner in leaf, but not in the culm. *ScGAI*-overexpressing transgenic sugarcane lines exhibited a stunted growth, shorter internodes, and impaired energy metabolism. In contrast, *ScGAI*-silenced plants were taller, with rapid internode elongation, increased phytomer production, and greater carbon allocation to the stem. The present study clearly shows a key regulatory role for ScGAI in sugarcane culm and leaf development and provides further insight into strategies for genetic improvement of this important food and fuel crop.

## Materials and methods

As an overview of our strategy to understand the role of ScGAI, our first approach was to molecularly characterize the *ScGAI* gene at different levels using a variety of techniques such as cloning, sequencing, and analysis of protein structure, phylogenetic diversity, expression patterns, subcellular localization, post-translational modification, and protein–protein interaction. In order to effectively address the evolutionary conservation of ScGAI function, we also overexpressed its coding sequence in transgenic Arabidopsis lines. Subsequently, to broaden our knowledge of how GAs modulate sugarcane growth, endogenously active GA levels were quantified, along with the expression levels of the gene encoding a key GA biosynthesis enzyme along the stem. Finally, for the functional characterization of *ScGAI*, transgenic sugarcane lines overexpressing and silencing the gene were screened for gene expression, cellular and phenotypic variations, and physiological, hormonal transcriptomic, and metabolomics changes.

### Plant material and growing conditions

Experiments were conducted with an Australian commercial sugarcane variety, Q208A, and two Arabidopsis ecotypes (Col-0 and Ler-0). All sugarcane plants used were grown in PC2 glasshouses at Sugar Research Australia, Brisbane. They were grown individually in pots (20 cm diameter) containing a 3:1 (v/v) soil and perlite (Chillagoe Perlite, Mareeba, Qld, Australia) potting substrate. Plants were fertilized monthly with Osmocote granules (Scotts Australia Pty Ltd, Australia) and irrigated for 30 s every 2 h between 06.00 h and 18:00 h daily using drip irrigation. The experiment followed a randomized complete block design with eight replicate blocks.

### Gene cloning and bioinformatics analyses

In order to characterize the sugarcane DELLA protein and to investigate its protein–protein interactions, the coding sequences of sugarcane genes *ScGAI* (accession no. MG766280), *phytochrome-interacting factor 3* (*ScPIF3*; accession no. MG766281), and *ethylene insensitive 3* (*ScEIN3*; accession no. MG766284), the genes reported to be interacting with DELLA in controlling growth, were amplified using specific primers from genomic DNA of sugarcane variety SP80-3280. Similarly, the cDNA clones of *ScPIF4* (accession no. MG766282), *ScPIF5* (accession no. MG766283), and *ethylene insensitive-like 1* (*ScEIL1*; accession no. MG766285) were also isolated and their coding sequences were amplified using specific primers (Supplementary Table S1 at *JXB* online). For evolutionary analysis, DELLA protein sequences from sorghum SbD8 (Sb01g010660), maize ZmD8 (Q9ST48) and ZmD9 (Q06F07), pea CRY (B2BA71) and LA (B2BA72), tomato LeGAI (Q7Y1B6), grape VvGAI (Q8S4W7), barley SLN1 (Q8W127), wheat Rht-1 (Q9ST59), rice SLR1 (Q7G7J6), and Arabidopsis AtRGL1 (Q9C8Y3), AtRGL2 (Q8GXW1), AtRGL3 (Q9LF53), AtGAI (Q9LQT8) and AtRGA (Q9SLH3) were aligned using the ClustalX program (Larkin *et al.*, 2007) and a phylogenetic tree was constructed using the neighbor-joining method (Saitou and Nei, 1987) available in MEGA6 (Tamura *et al.*, 2013) with bootstrap analysis of 1000 replicates. Predicted tertiary structure of sugarcane DELLA domain was obtained with the SWISS MODEL program (Biasini *et al.*, 2014). The X-ray crystal structure of Arabidopsis AtGAIn-AtGID1A/GA3 complex (PDB entry 2zsh.1.b) was used as a model.

### Recombinant ScGAI protein expression in *Escherichia coli*

Full-length *ScGAI* was isolated using specific primers (Supplementary Table S1) and cloned into pET21a(+) vector (Novagen, USA). The His-tagged ScGAI protein was expressed in *Escherichia coli* BL21 (DE3) strain after 4 h of induction with 1 mM isopropyl β-D-1-thiogalactopyranosideat 37 °C. Both cell extracts (soluble and insoluble fractions) were analysed by SDS-PAGE and western blotting.

### Protein expression pattern

Total protein was extracted according to the phenol protocol (Amalraj *et al.*, 2010) and quantified by Bradford reagent (Bio-Rad, USA). Equal amounts of total protein were separated in NuPAGE Novex 4–12% gradient Bis-Tris gel, transferred onto polyvinylidene fluoride (PVDF) membranes and probed with polyclonal antibody raised (1:1000 dilution) against the N-terminus of sugarcane DELLA (anti-ScGAI). Secondary horseradish peroxidase-conjugated anti-rabbit IgG was used at a dilution of 1:1000. Immunoblotted bands were visualized by the SuperSignal West Pico Chemiluminescent substrate (Pierce). PVDF membranes were stained with Coomassie Blue.

### Subcellular localization

The *ScGAI*, *ScPIF3*, *ScPIF4*, *ScEIN3*, *ScEIL1*, and *gai∆Nterminal* coding regions were cloned in frame with VENUS protein into the pART7 vector (Gleave, 1992) and transiently expressed in Arabidopsis protoplasts using pART7:VENUS as a positive control. Each construction was transfected with the vector pART7:AtPARP3:mCherry, a positive nuclear control (Rissel *et al.*, 2014). The images were captured with an AxioCam MRM Observer Z1 Zeiss AX10 microscope (Zeiss, Germany).

### Protein–protein interaction studies

The coding sequences of the *ScGAI* and *gaiΔdella* (201–625) were cloned into pGBKT7 vector, while the coding sequences of *ScPIF3*, *ScPIF4*, *ScPIF5*, *ScEIN3*, *Scein3*(233–552) and *ScEIL1* were cloned into pGADT7 vector. These vectors were introduced into yeast strain Y2HGOLD following protocol in the Matchmaker Gold Yeast Two-Hybrid System user manual (Clontech, Takara Bio Inc., Japan).

For biomolecular fluorescence complementation (BIFC) assay, protoplasts isolated from 3- to 4-week-old Arabidopsis Col-0 leaves were used for DNA transfection. To generate N-terminal and C-terminal yellow fluorescent protein (YFP)-tagged constructions, the coding regions of *ScGAI*, *ScPIF3*, *ScPIF4*, *ScEIN3*, and *ScEIL1* were amplified using specific primers (Supplementary Table S1) and subcloned into pGEMTEasy, and then cloned into the pUC_SPYNE vector. N-terminal truncated *ScGAI* was cloned into the same vectors and used as negative control. The plasmids were co-transfected into freshly prepared Arabidopsis leaf mesophyll protoplasts (Yoo *et al.*, 2007). Images were captured using an AxioCam MRM Observer Z1 Zeiss AX10 microscope (Zeiss, Germany).

### SUMOylation analysis

For immunoprecipitation assay, total protein extracts were prepared from sugarcane leaf +1. Protein extracts (300 µg) were incubated with 3 µg of anti-SUMO1 polyclonal antibody (Abcam; ab5316) bound to anti-rabbit IgG-coated magnetic beads (Dynabeads M-280 sheep anti-rabbit IgG; Invitrogen) for 1 h at room temperature. Subsequent washing steps were performed with phosphate-buffered saline and the target antigen was eluted with NuPAGE LDS sample buffer. Immunoprecipitated small ubiquitin-like modifier (SUMO)–ScGAI proteins were detected by immunoblot analysis using anti-ScGAI (GenScript). SUMO1-coupled Dynabeads incubated with protein extraction buffer served as negative control.

### Functional analysis of ScGAI in transgenic Arabidopsis

The binary vector pGREENII0179 harboring the *hyg* gene was used for overexpressing the *ScGAI:VENUS* coding region in Arabidopsis ecotype Ler-0. The pGREENII:ScGAI:VENUS and pGREENII vectors were introduced into *Agrobacterium* GV3101 strain by electroporation. The helper plasmid pSOUP was introduced with each vector, to provide the replicase gene (*RepA*) for pGREEN vector replication. Plants were transformed following the floral dip protocol (Logemann *et al.*, 2006) and T1 seeds obtained were plated onto Murashige and Skoog (MS) medium containing 20 mg l^−1^ of hygromycin.

### Measurement of GAs

Internode samples from apical shoot (Z1), and elongating (Z2), maturing (Z3), and mature (Z4) internodal zones were flash frozen in liquid nitrogen, ground, lyophilized, and analysed for bioactive GAs at the Proteomics and Metabolomics Facility, Center for Biotechnology, University of Nebraska – Lincoln as described in Hung *et al.* (2016).

### Functional analysis of ScGAI expression in transgenic sugarcane plants

The young unfurled three to four innermost leaves covering the shoot apex from a sugarcane commercial variety (*Saccharum* spp. L. var. Q208^A^) were isolated under sterile condition and used for biolistic transformation as described previously (Joyce *et al.*, 2014). For this procedure, gold particles were coated with a 1:1 molar ratio of plasmids pUbi:FLAG:ScGAI or pUbi:hpGAIi (see Supplementary Fig. S4 for more details about the transgenes) and pUKN (for geneticin-based plant selection). All regenerated plants were maintained on geneticin selection medium under 16 h photoperiod at 28 ± 1 °C in a PC2 plant culture room until they were transferred to a PC2 glasshouse as potted plants. For all experiments, plants taken through the transformation procedure but without introducing transgene served as control.

### Molecular characterization of transgenic sugarcane with altered ScGAI

Genomic DNA from transgenic sugarcane leaves was extracted as previously described (Aljanabi and Martinez, 1997). PCR genotyping to detect the presence of the transgene was performed using a different set of primers (Supplementary Table S1) following the protocol of GoTaq Green Master Mix (Promega, USA).

### Gene expression analyses

Leaf tissue samples were flash frozen and ground with liquid nitrogen to a fine powder in a Precellys 24 mini-bead beater (Bertin Technologies, France) and high-quality total RNAs were isolated and purified according to the Spectrum Plant total RNA kit protocol (Sigma-Aldrich, USA).

For cDNA synthesis, total RNA was treated with RQ1 RNase-Free DNase (Promega, USA) at 37 °C for 30 min to remove genomic DNA contamination, which was confirmed by PCR. Full-length cDNAs were synthetized with Improm-II reverse transcriptase enzyme (Promega, USA). Each reaction mixture contained 5 µl of SensiMix SYBR Low-ROX (Bioline, Australia), 0.2 µl (200 nM) of gene-specific forward and reverse primers and 1.6 µl water. An epMotion M5073 liquid handler (Eppendorf) was used to aliquot the reagent mix and 3 µl of 5 ng µl^−1^ cDNA into MicroAmp® Fast Optical 384-Well Reaction Plates (Life Technologies, Australia). The thermal profile was 95 °C for 10 min, 40 cycles of 95 °C for 15 s and 60 °C for 50 s, followed by a dissociation step of 95 °C for 2 min, 60 °C for 15 s. All qPCR data generated were analysed using DataAssist™ Software (Life Technologies, Australia). For each cDNA sample, an average gene amplification level was calculated from triplicate PCR reactions (technical replicates). This average expression for each gene was normalized against the average expression level of a reference gene (actin depolymerizing factor; ADF), to account for template variations between samples. Then each expression level was compared with a reference sample according to the $2^{-\Delta \Delta C_{\text{q}}}$ method (Livak and Schmittgen, 2001).

### Histological and phenotypic analysis

Transverse cross-sections of leaves and stem of 3-month-old transgenic and control plants grown in the glasshouse were stained with 0.05% Toluidine Blue or with 1% (w/v) phloroglucinol in 1 M HCl (for lignin) for 30 s. After washing, the stained sections were photographed with an Olympus DP70 (Olympus America Inc., USA) camera. For phenotypic analysis, stem height was measured with a ruler from the soil, and the internode diameter was measured with a digital caliper.

### Effect of gibberellin (GA_3_) and paclobutrazol on transgenic sugarcane

Transgenic and control sugarcane plantlets were transferred to large sterilized jars containing 100 ml of MS medium (pH 5.8) with or without 50 µM GA_3_ (Phytotechnology Laboratories, USA) or 5 µM paclobutrazol (PAC; Phytotechnology Laboratories, USA). Plants were grown in a growth room maintained at 28 °C with a 16/8 h light/dark cycle for 23 d and were phenotyped.

### Sugarcane leaf starch assay

Leaf discs were collected from 4-month-old control and transgenic sugarcane plants grown in the glasshouse, depigmented with ethanol, and rinsed with distilled water. Depigmented samples were stained with 1% Lugol’s IKI solution at room temperature for 5 min and rinsed with distilled water. Images of stained leaves were captured with a Sony DSC-HX200V digital camera. The enzymatic starch assay was concurrently performed as previously described (Marquardt *et al.*, 2016).

### Photosynthesis measurements

Photosynthetic parameters were measured using intact sugarcane leaf +1 of 5-month-old glasshouse-grown transgenic and control plants with LI-COR infrared gas analyser LI-6400 (LI-COR Bioscience, USA). The chamber light (photosynthetically active radiation) level was set to 2000 µmol photons m^−2^ s^−1^ and reference CO_2_ to 400 µmol mol^−1^.

### Illumina sequencing

Total RNA from leaf +1, apical shoot, and fifth and ninth internodes from 6-month-old transgenic and control plants was extracted following the Spectrum Plant total RNA kit protocol (Sigma-Aldrich). Extracts were treated with RQ1 RNase-Free DNase (Promega, USA) according to the manufacturer’s protocol. For each tissue, pooled RNA samples from four biological replicates were used for analysis. One microgram of each RNA sample was used to produce cDNA libraries, which were sequenced using an Illumina HiSeq 2500 by Fasteris Life Science Co. (Geneva, Switzerland). The reads were mapped on *Sorghum bicolor* reference genome available at Illumina iGenomes (http://support.illumina.com/sequencing/sequencing_software/igenome.html). The list of differentially expressed genes (DEGs) was identified using an FDR *q*-value cutoff of 1 × 10^−5^. The sequence data were deposited in the NCBI SRA database with the accession number SRP132098 (http://www.ncbi. nlm.nih.gov/sra/SRP132098).

### Metabolite profile analysis

Five milligrams of leaf, shoot apex, and fifth and ninth internodal tissues from four biological replicates (6-month-old transgenic and control plants) was snap frozen in liquid nitrogen, ground to a fine powder and lyophilized prior to extraction using MTBE:methanol:water 3:1:1 (v/v/v). The organic phase (100 μl) was dried and derivatized. One microliter of derivatized sample was analysed using a Combi-PAL autosampler (Agilent Technologies GmbH, Waldbronn, Germany) coupled to an Agilent 7890 gas chromatograph connected to a Leco Pegasus 2 time-of-flight mass spectrometer (LECO, St Joseph, MI, USA). Chromatograms from Leco ChromaTOF (version 3.25) software were exported to R software. Peak detection, retention time alignment, and library matching were performed using the Target Search R-package (Cuadros-Inostroza *et al.*, 2009). Metabolites were quantified by the peak intensity of a selective mass. Metabolite intensities were normalized by dividing by the fresh weight, followed by the sum of total ion count and global outlier replacement. Principal component analysis was performed using the pcaMethods bioconductor package (Stacklies *et al.*, 2007). Statistical significance of metabolite variation was determined by comparing the data from a given tissue from all genotypes by Tukey’s test.

## Results and discussion

### *ScGAI* encodes a DELLA protein

**Fig. 1.**
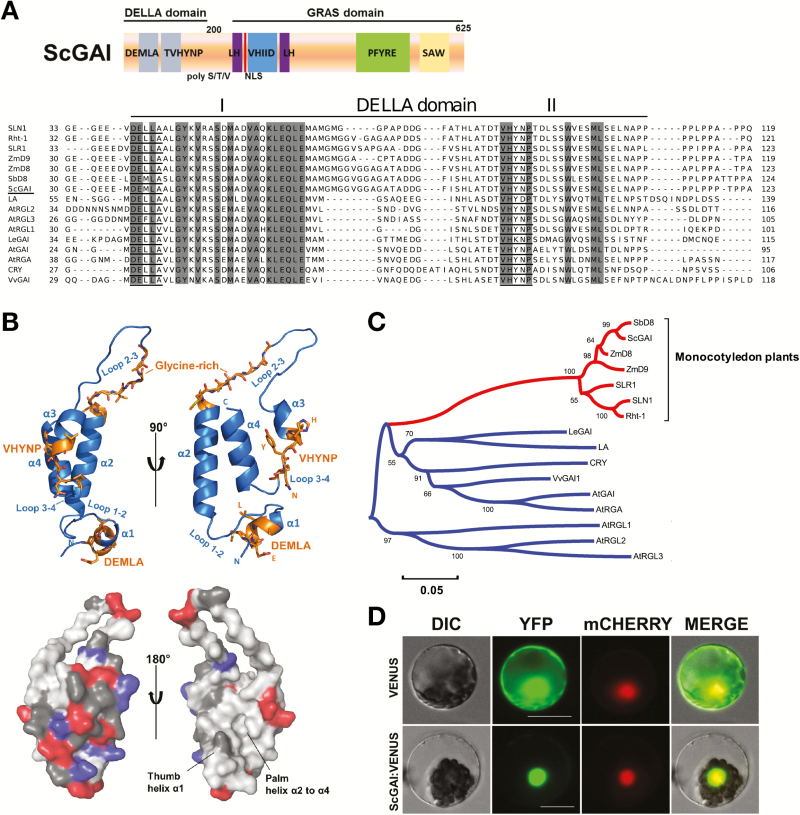
*ScGAI* encodes the nuclear DELLA protein in sugarcane. (A) Top, schematic drawing of ScGAI protein showing all the conserved domain along the sequence; bottom, protein alignment of DELLA domain highlighting the identical amino acids among the sequences. DELLA and TVHYNP amino acids are underlined. (B) Cartoon (top) and surface (bottom) representation of the predicted tertiary structure of DELLA domain from ScGAI. The electrostatic surface is represented by regions negatively charged (red), positively charged (blue), polar (dark gray) and hydrophobic (light gray). The overlap between predicted and native structure has a root-mean-square deviation (RMSD) value of 0.091. (C) Phylogenetic tree of DELLA family in Arabidopsis, tomato, pea, grape, barley, wheat, rice, maize, sorghum, and sugarcane. The red branch of the tree is the conserved DELLA family in monocotyledonous plants. (D) Subcellular localization of ScGAI expressed in Arabidopsis mesophyll protoplast. The construct *AtPARP3:mCHERRY* (mCHERRY) was used as nuclear control. DIC, differential interference contrast; YFP, yellow fluorescent protein. Scale bars, 20 µm.

Using the Brazilian SUCEST project database (http://www.sucest-fun.org/), we have identified and cloned the *ScGAI* gene in sugarcane. The *ScGAI* gene presents an open reading frame of 1878 bp and encodes a protein with 625 amino acid residues. The *ScGAI*-deduced amino acid sequence contains all conserved regions of DELLA proteins, including the N-terminal DELLA regulatory domain that contains the DELLA, TVHYNP, and poly S/T/V motifs and a C-terminal GRAS domain that comprises the leucine heptad repeats (LHI and LHII) that flank the VHIID motif, and the PFYRE and SAW motifs (Fig. 1A). However, notably, ScGAI exhibited an L39M change (DEMLA) within the DELLA motif, which is also conserved in the SbD8 protein from sorghum. Likewise, the Arabidopsis DELLA protein AtRGL3 also shows an L36F change in this region, which does not prevent its interaction with AtGID1s receptors (Nakajima *et al.*, 2006). Although the hydrophobic DELLA and TVHYNP motifs are important for interacting with GA receptors and affecting GA-dependent DELLA degradation (Murase *et al.*, 2008), a recent study confirmed that the DELL amino acid residues are not required for this interaction (Sheerin *et al.*, 2011). The predicted tertiary structure of the DELLA domain from ScGAI showed high molecular spatial similarity to the solved domain structure of the AtGAI protein from Arabidopsis (Fig. 1B). Nevertheless, it is worth noting the presence of a glycine-rich region (G^67^MGGVGG^73^) encompassing amino acids within the loop 2–3 in ScGAI protein. The sequence GMGG seems to be specific for monocot DELLA proteins. Glycine-rich loops or P-loops are known to function as ATP-binding pockets (Saraste *et al.*, 1990). Phylogenetic analysis revealed that ScGAI is highly homologous to SbD8 and ZmD8 proteins in sorghum and maize, respectively. The evolutionary divergence of DELLA proteins in monocot plants is clearly lower in comparison with dicotyledonous plants (Fig. 1C), evidencing that *DELLA* genes are under differential selective pressures between dicot and monocot plants.

DELLA proteins are known to be nuclear transcriptional regulators. To determine the subcellular location in sugarcane, ScGAI was transiently expressed in Arabidopsis mesophyll protoplasts cells. ScGAI was found to be localized in the nucleus (Fig. 1D), corroborating the presence of a putative SV40-like sequence (K^182^RMK^185^) before the poly-S/T/V region and one well-defined bipartite NLS sequence (R^281^KVAAYFGEALARR^294^) localized in the LH domain. We then asked whether ScGAI is indeed a functional protein involved in GA signaling. As shown in Supplementary Fig. S1, overexpression of *ScGAI* in Arabidopsis repressed GA responses such as rosette diameter and stamen development, phenotypes that are also observed in the dominant GA-insensitive Arabidopsis *gai-1* mutant (Cheng *et al.*, 2004). Collectively, these results demonstrated that ScGAI acts as a bona fide DELLA protein.

### Expression of *ScGAI* and bioactive GAs are spatially regulated in sugarcane culm

**Fig. 2.**
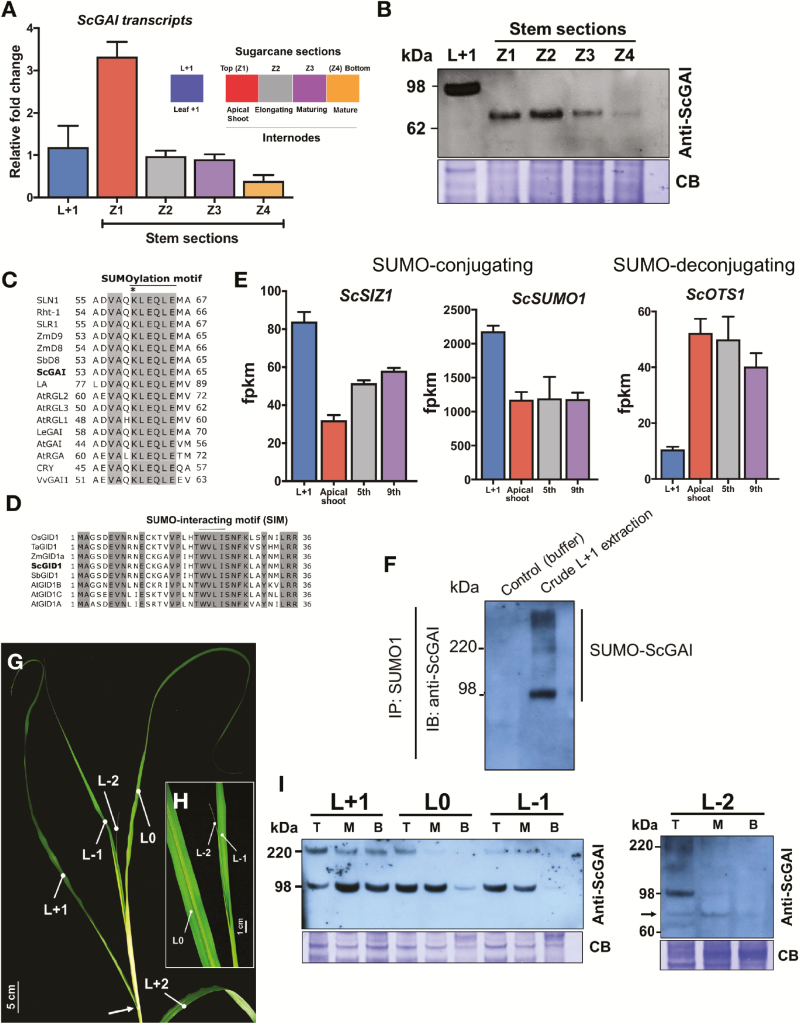
ScGAI is SUMOylated in sugarcane leaves. (A) Expression profile of native *ScGAI* in different tissues of 10-month-old sugarcane; bar plots show means ±SD of three biological replicates. (B) Immunoblotting of ScGAI protein in sugarcane tissues. (C) Sequence alignment of the non-canonical SUMOylation motif in DELLA proteins. Asterisk represents the conserved SUMOylation site lysine residue. (D) Sequence alignment of GID1 from rice, wheat, maize, sorghum, Arabidopsis, and sugarcane displaying the SUMO-interacting motif (SIM). Light gray depicts the conserved amino acids among the sequences. (E) Expression profile analysis of *ScSIZ1*, *ScSUMO1*, and *ScOTS1* transcripts in leaf +1 (L+1), apical shoot, and fith and ninth internodes. FPKM, fragment per kilobase of exon per million fragments mapped. Bars show means ±SD of three biological replicates. (F) Immunoprecipitation using anti-SUMO1 antibodies in crude extract of leaf +1. (G) The leaf numbering system proposed by Kuijper (1915). The first fully expanded leaf with visible dewlap (indicated by an arrow) and photosynthetically active was considered as leaf +1. (H) Close-up view of juvenile leaf L−2. (I) Immunoblotting of the ScGAI protein in different sections of juvenile (L0, L−1 and L−2) and fully expanded (L+1) leaves of Q208 (1 month old). The arrow indicates the non-SUMOylated ScGAI protein. Equal amounts of protein samples (10 µg) were loaded. CB, Coomassie Blue-stained membrane as loading control. B, base; M, middle; T, tip.

In sugarcane, *ScGAI* showed the highest expression level in the shoot apical meristem (SAM) (Fig. 2A), as observed in Arabidopsis, tomato, and rice (Schmid *et al.*, 2005; Jain *et al.*, 2007; Jasinski *et al.*, 2008). Western-blot analysis showed that the ScGAI protein is highly abundant in the SAM and is also present in elongating internodes of sugarcane culm (Fig. 2B). In contrast to ScGAI, bioactive GAs were found to be lowest at the shoot apical region with their content increasing basipetally, reaching the highest values in basal mature internodes (Supplementary Fig. S2). Among the bioactive GAs, namely GA_1_, GA_3_, and GA_4_, only GA_3_ was detected at a higher concentration in the mature internodes. GA_3_ is formed from GA_20_ using the intermediate GA_5_, which is present in several monocotyledons (Hedden and Thomas, 2012). It is therefore possible that the expression of *ScGA20ox*, a GA biosynthesis enzyme, is up-regulated by sucrose in sugarcane, leading to increased GA production in the mature internodes (Liu *et al.*, 2011) (Supplementary Fig. S2). The role of bioactive GAs in increasing hydrolase activity might explain the biological significance of high GA_3_ content in mature internodes. Further studies are needed to elucidate the synthesis, transport, and action of GAs in sugarcane.

### ScGAI is a regulatory component of spatio-temporal leaf growth in sugarcane, and its action is modulated by SUMOylation

ScGAI was present in leaf +1, the youngest fully expanded leaf, with an estimated molecular mass of 98 kDa (Fig. 2B), which is higher than the predicted mass of 66 kDa for ScGAI found in the stem tissues and also in His-tagged ScGAI expressed in *E. coli* (Supplementary Fig. S3). Further analysis showed that ScGAI has a small ubiquitin-like modifier (SUMO) protein binding site, a SUMOylation motif (Fig. 2C), within the DELLA domain, suggesting possible SUMOylation of leaf ScGAI. SUMO interacts with DELLA proteins through a covalent binding in the N-terminal DELLA domain. SUMOylated DELLA interacts with the GA receptor GID1 through the SUMO-interacting motif (SIM) in a GA-independent manner (Conti *et al.*, 2014). This SUMO–SIM interaction sequestrates GID1, blocking its access to the DELLA domain and consequently preventing the GA-triggered DELLA degradation. In our study, SIM was found in the sugarcane GA receptor ScGID1 (Fig. 2D). Moreover, expression profile analysis of the E3 SUMO ligase *ScSIZ1* and *ScSUMO1* genes involved in the covalent SUMO conjugation process showed a significantly higher level of expression in leaf +1 in comparison with internodes in sugarcane (Fig. 2E). On the other hand, the expression level of the SUMO protease OVERLY TOLERANT TO SALT1 (*ScOTS1*) gene, whose product mediates the deconjugation of SUMO, was drastically reduced in leaf +1 (Fig. 2E). To confirm the SUMOylation of ScGAI, protein lysates were immunoprecipitated using anti-SUMO1 antibodies, which proved that the high molecular mass band observed in leaf sample was indeed the SUMOylated ScGAI (Fig. 2F). In order to obtain more insights into the SUMOylation of ScGAI in leaves, tip, middle, and basal sections of young developing juvenile (0, −1 and −2) and fully expanded (+1) leaves were analysed (Fig. 2G). ScGAI was SUMOylated in the mature tissues of the juvenile and fully expanded leaves and this was gradually reduced in the middle and basal sections of young developing leaves, where cell elongation and division still occur (Fig. 2I). Taken together, the results indicate that sugarcane leaf growth may be controlled by SUMOylation of ScGAI in a spatio-temporal manner.

### *ScGAI* expression determines sugarcane growth and morphology

**Fig. 3.**
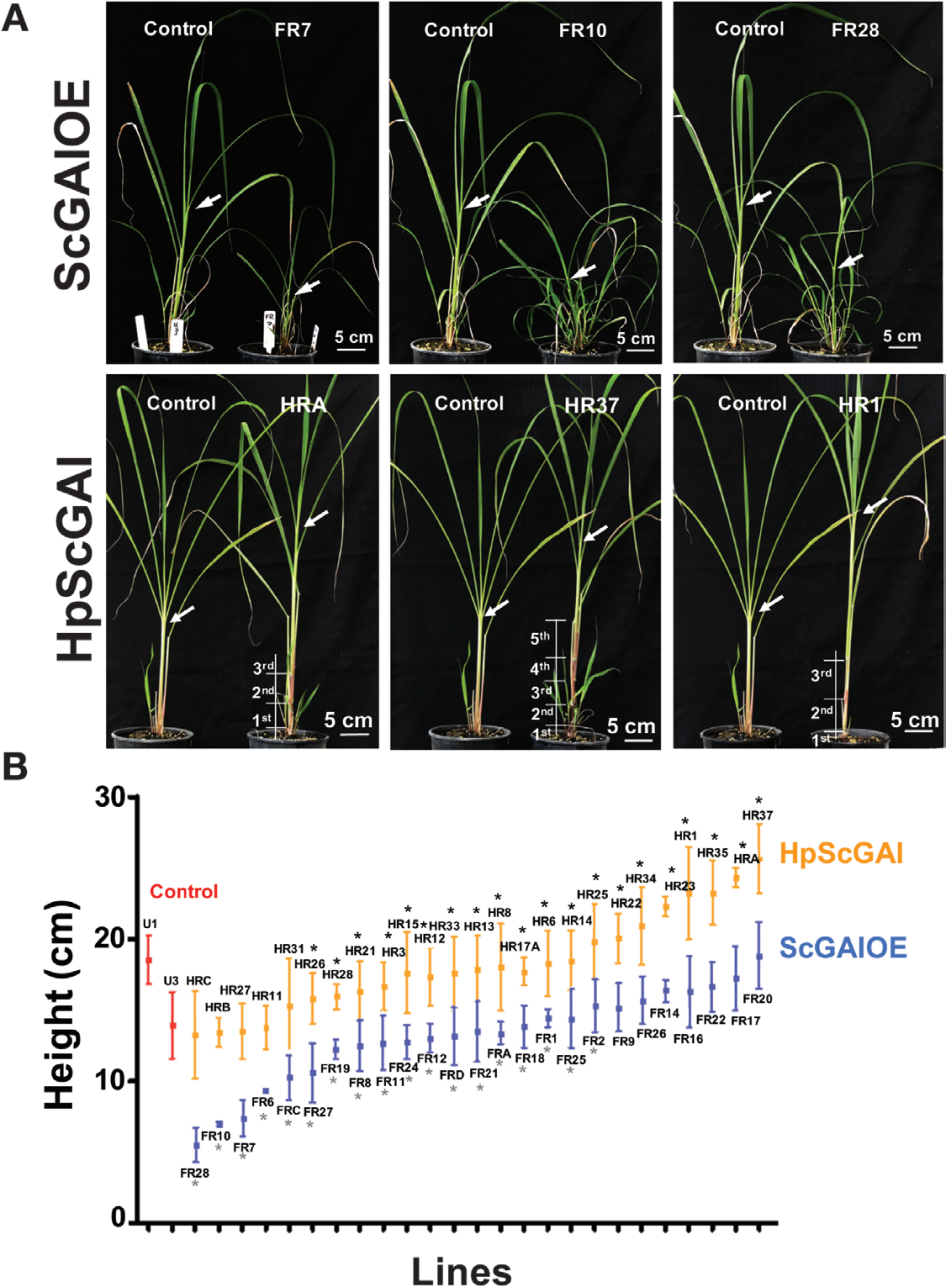
*ScGAI*-misexpressing sugarcane lines. (A) Phenotype of ScGAIOE and HpScGAI lines showing the stunted and taller stems, respectively. The earlier onset of elongated internodes in HpScGAI is numbered from the soil to the top. Arrows indicate the first visible dewlap. (B) Height of 3-month-old sugarcane plants. The data points represent means ±SD of three biological replicates. Red lines U1 and U3 represent untransformed control plants. Untransformed control plants were produced through all the tissue culture and transformation steps used for generating transgenic plants but without the introduction of transgene. The group comprising all HpScGAI lines exhibited significantly higher values for height compared with the group of ScGAIOE lines (unpaired one-tailed *t*-test, *P*<0.01). The gray asterisks indicate significance (*P*<0.05) for unpaired one-tailed *t*-test between the group comprising ScGAIOE lines FR28 to FR2 and untransformed control plants; the black asterisks indicate significance (*P*<0.05) for the comparison between the group comprising HpScGAI lines HR26 to HR37 and untransformed control plants.

**Fig. 4.**
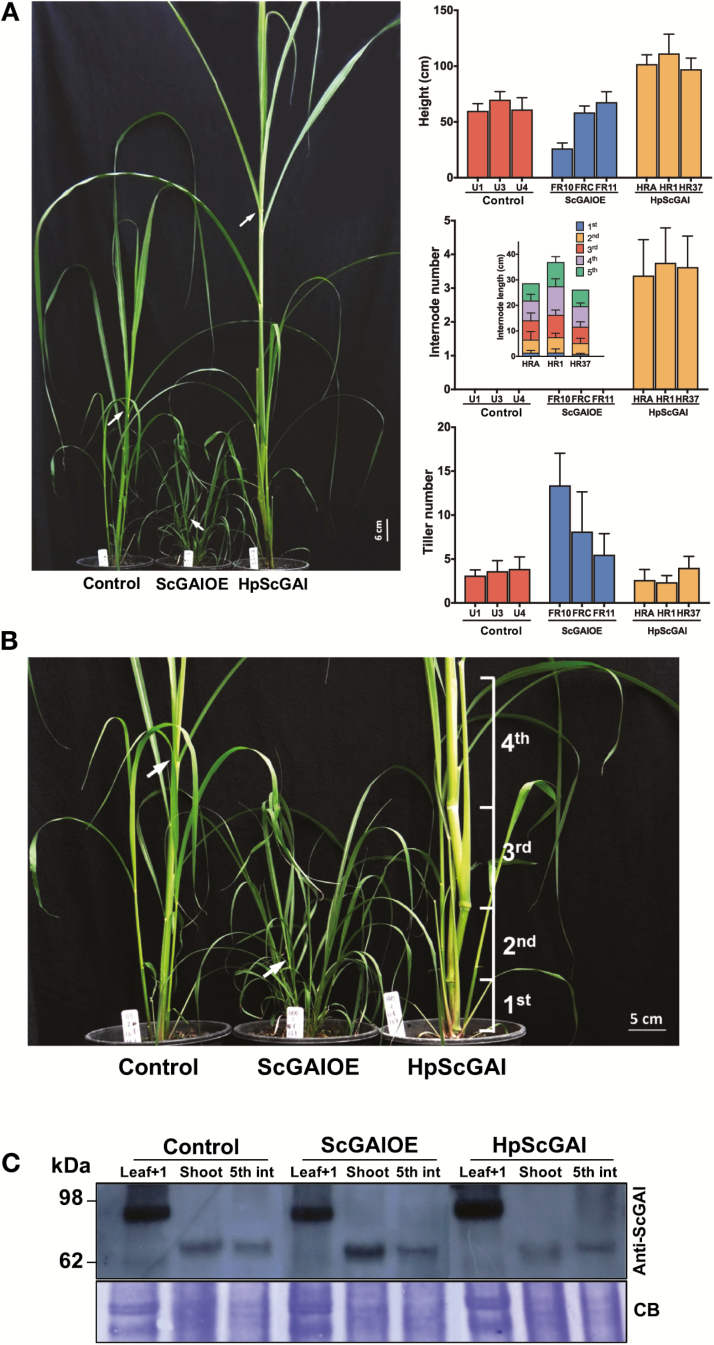
ScGAI regulates tillering and culm development in sugarcane plants. (A) 3-month-old transgenic FR10 (ScGAIOE; dwarf), HR1 (HpScGAI; tallest), and untransformed lines. Height, internode number, and elongation and tiller number; bars show means ±SD of eight biological replicates. (B) Zoomed-in detailed view of 3-month-old plants. Internode numbers counted from soil to top. Arrows indicate the first visible dewlap. (C) Immunoblotting using a sugarcane anti-DELLA (anti-ScGAI) antibody. Each lane was loaded with 20 µg of total protein from leaf +1, apical shoot (shoot) and fifth internode (5th int) tissues of untransformed control, FR10, and HR1 lines of 6-month-old plants; CB, Coomassie Blue.

To elucidate the role of ScGAI in sugarcane, transgenic lines with up-regulated (ScGAIOE) or down-regulated (HpScGAI) *ScGAI* expression were generated (Supplementary Fig. S4). ScGAIOE lines displayed a high *ScGAI* transgene expression level, while the opposite was true for HpScGAI lines (Supplementary Fig. S5). As shown in Fig. 3A, B, the *ScGAI* transgenic lines displayed a range of growth and developmental variations. Culm growth was the most affected function with altered *ScGAI* expression (Fig. 4B). The extreme phenotypes could be clearly distinguished from control plants, showing a highly stunted stature with high tillering among ScGAIOE lines, while there was a taller stature with early onset of visible nodes and internodes in HpScGAI plants (Figs 3A, 4A, B; Supplementary Fig. S6). It is worth noting that HpScGAI plants showed no changes in stem diameter compared with control throughout their development (Supplementary Fig. S6). In addition, we observed no changes in ScGAI protein content in leaf tissues from transgenic plants (Fig. 4C). As described above, SUMOylation seems to coordinate sugarcane leaf development through appropriate spatio-temporal stabilization of ScGAI.

To assess whether changes in the morphology were accompanied by anatomical variation, cross-sections of leaves and stem of transgenic and control plants were studied (Supplementary Fig. S7). We did not observe any anatomical differences in the leaves. However, as expected, culm development was pronounced with accelerated phytomer production in HpScGAI lines even in 3-month-old plants, such that they had a well-developed culm ground tissue composed of storage parenchyma cells and vascular bundles in their culm, while such structural/anatomical features were not evident in control and ScGAIOE plants. In agreement with this fast culm development rate, lignification of basal internodes was observed in *ScGAI*-silenced plants (Supplementary Fig. S7). These results clearly demonstrated a strong regulatory role for ScGAI in culm growth and development and tillering in sugarcane.

### Transgenic sugarcane with altered *ScGAI* expression is hypersensitive to GA and paclobutrazol

To gain further insight into the functional role of GA/ScGAI regulation in sugarcane, HpScGAI and ScGAIOE lines and control plants were treated with GA_3_ (50 µM) or PAC (5 µM), an inhibitor of GA biosynthesis (Supplementary Fig. S8). Following the PAC treatment, control plants produced a very short and thick culm. HpScGAI plants, however, were less sensitive to PAC compared with control, indicating diminished DELLA repressor activity. On the other hand, ScGAIOE plants showed a stronger response to PAC than the control. Strikingly, all PAC-treated plants showed increased root growth, though to a lesser degree in ScGAIOE lines. As expected, GA_3_ treatment rescued the short phenotype of ScGAIOE plants but made HpScGAI plants highly slender and taller with twice the height of GA_3_-treated control (Supplementary Fig. S8). These results further demonstrate that GA and DELLA play a central role in culm growth and development and modulation of shoot-to-root ratio in sugarcane.

### Change in *ScGAI* activity causes a large shift in gene expression

The remarkable organ-specific developmental variations caused by the change in *ScGAI* activity in sugarcane point towards a major shift in the expression of key genes underpinning the observed phenotypes. Analysis of gene expression by RNA sequencing (RNA-seq) identified DEGs in leaves and internodes of ScGAIOE and HpScGAI plants. Overall, 345 DEGs showed a statistically significant difference between the lines FR10 (ScGAIOE; dwarf line) and HR1 (HpScGAI; tallest line) (Supplementary Fig. S9; Supplementary Tables S2–S5). In both plants, the highest number of DEGs was found in the stem, mainly in the ninth internode, the maturing tissue. Among the DEGs related to growth, three ethylene-responsive element binding factor (ERF) genes were expressed at high levels in HpScGAI in elongating tissues. ERF genes are known to be involved internode elongation, and they are well characterized in rice (Hattori *et al.*, 2009). Besides ERF genes, *Brz-insensitive-long hypocotyls 4* (*BIL4*), another positive regulator of plant cell elongation via brassinosteroid signaling (Yamagami *et al.*, 2009), had higher expression in HpScGAI plants. This indicates that a cross-talk between GA, DELLA, and other growth-related hormones is affecting the observed growth and developmental variations. On the other hand, overexpression of *ScGAI* in ScGAIOE activated several genes related to sucrose transporter, energy metabolism, and stress responses, such as the Snf1-related kinase 1 (SnRK1) regulatory subunit *KINβ1*, two key regulators of the starvation response, such as the *basic region-leucine zipper transcription factor 63* (*bZIP63*) and *dark-inducible 6* (*DIN6*), as well as the trehalose-6-phosphate synthase genes *TPS9* and *TPS11*, which are involved in trehalose biosynthesis. All these genes form a network regulating metabolism under stress conditions in order to preserve energy. The transcriptomic data presented suggest the proposition that ScGAI regulates a complex transcriptional network of genes to modulate growth, energy metabolism, and possibly stress responses in sugarcane.

### ScGAI-mediated culm growth regulation modulates source–sink relationship in sugarcane

**Fig. 5.**
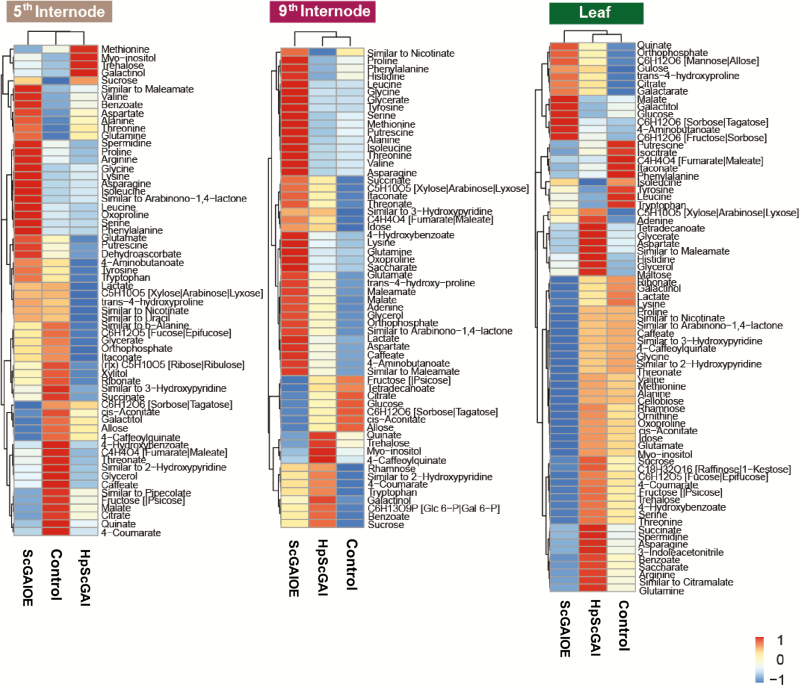
Carbon balance is severely altered in transgenic plants. Metabolite-based clustering of leaves (L+1) and fifth and ninth internodes in ScGAIOE (FR10 line) and HpScGAI (HR1 line) compared with untransformed control. The intensities are color-coded. Red represents high and blue represents low intensities. The statistical significance of metabolite variation was determined by comparing the data from a given tissue from all genotypes by Tukey’s test (Supplementary Dataset S1).

In order to understand the carbon homeostasis in the transgenic plants, we analysed the leaf (L+1) and internode (fifth and ninth) metabolome of 6-month-old plants (Fig. 5). There were significant changes in sugar and amino acid levels in ScGAIOE leaves, while there were relatively smaller changes in the leaves of HpScGAI lines, compared with control (Fig. 5). In agreement with this, the rate of photosynthesis and sucrose levels were significantly reduced in ScGAIOE lines (Supplementary Fig. S10). However, surprisingly, malate content rose to high levels in ScGAIOE background (Fig. 5), as observed in PAC-treated Arabidopsis (Ribeiro *et al.*, 2012). This result led us to suspect a change in the diurnal rhythm of starch accumulation in ScGAIOE leaves. At dusk, leaves of ScGAIOE plants contained much less starch than HpScGAI and control plants (Supplementary Fig. S11), and this observation was further supported by the results of enzymatic assays. At the sink level (in culm), a high accumulation of amino acids was observed in the fifth and ninth immature internodes, possibly due to limited demand for growth (Fig. 5). On the other hand, the investment of carbon into storage molecules and phenylpropanoid synthesis was markedly more evident in HpScGAI lines, with the level of metabolites such as sucrose, trehalose, galactinol, *myo*-inositol, and 4-caffeoylquinate being higher in both internodes (Fig. 5). Collectively, the results from metabolome and transcriptome analysis suggest a role for ScGAI in linking growth and primary metabolism in sugarcane.

### ScGAI interacts with ScPIF3/PIF4 and ScEIN3/ScEIL1 proteins to modulate growth

**Fig. 6.**
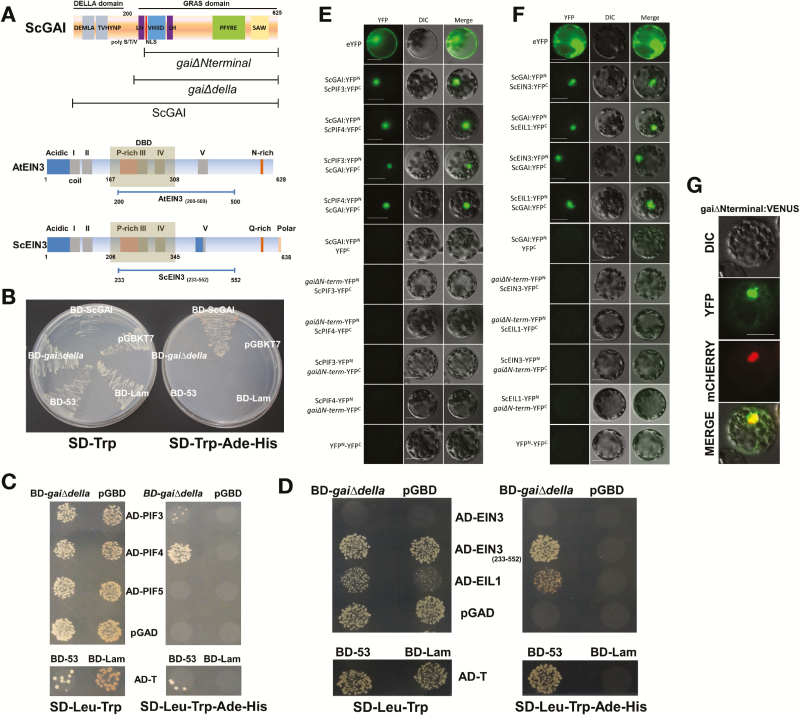
ScGAI interacts with ScPIF3/4 and ScEIN3/EIL1 in sugarcane. (A) Structure of the sugarcane DELLA ScGAI and its truncated versions used in the screening. Protein schematic comparison between AtEIN3 and ScEIN3 sequences and the protein truncation ScEIN3(233–522) used in the yeast two-hybrid assay. (B) Auto-activation activity of the different bait constructs in yeast cells. Full-length DELLA was capable of activating the transcription of reporter genes in the absence of prey proteins and also was toxic upon expression in yeast cells. (C, D) Co-transformations with different combinations were performed. On SD-Leu-Trp medium, diploid yeast cells were confirmed. On SD-Leu-Trp-Ade-His medium, only positive yeast cells for protein–protein interaction grew. AD, activation domain; BD, binding domain; pGBD and pGAD, empty vectors. Positive controls: 53-BD encodes murine p53 and T-AD encodes the SV40 large T-antigen. Negative control: Lam-BD encodes lamin. (E, F) BIFC assay was performed in Arabidopsis protoplasts. YFP^N^ and YFP^C^, N-terminal and C-terminal yellow fluorescent protein, respectively. (G) Subcellular localization of the truncated protein, namely gaiΔNterminal:VENUS, used as negative control in the BIFC assay. AtPARP3:mCHERRY was used as nuclear control. Scale bars, 20 µm.

Our next question was to find the molecular players directly associated with ScGAI in regulating sugarcane shoot growth. It is known that DELLA restrains growth through its interactions with PHYTOCHROME-INTERACTING FACTORS (PIF) proteins in Arabidopsis (de Lucas *et al.*, 2008). In this study, three sugarcane PIF protein-encoding genes (*ScPIF3*, *ScPIF4*, *ScPIF5*; Supplementary Fig. S12) were cloned and their interaction with ScGAI was investigated (Fig. 6). ScGAI was found to directly interact with ScPIF3 and ScPIF4, but not with ScPIF5 (Fig. 6C, E), demonstrating that the DELLA–PIF interaction seems to be conserved in both monocots and dicots. To further explore this ScGAI interaction network and to gain more mechanistic understanding of accelerated phytomer production in HpScGAI plants, we identified and cloned two key transcription factors of ethylene signaling, *ScEIN3* and *ScEIL1* (Supplementary Fig. S13), homologs of proteins known to modulate the expression of ERF proteins in Arabidopsis (Chang *et al.*, 2013). Interestingly, ScGAI interacted with both ScEIN3 and ScEIL1 (Fig. 6D, F), suggesting that ScGAI controls growth and development through a complex network, integrating external and endogenous signals.

### Sugarcane and the slow rate of genetic improvement

Plant hormones play a central role in integrating the external and internal cues and modulating growth. In sugarcane, where growth is considered to be strongly tempered by the source–sink relationship (McCormick *et al.*, 2009), our results clearly demonstrate the potential for manipulating source–sink control and thereby growth and development, through altered gibberellin action (Figs 3–5). This was achieved by modulating the activity of sugarcane gibberellin signaling inhibitor, ScGAI, which also showed gibberellin inhibitory activity in transgenic Arabidopsis (Supplementary Fig. S1). This strategy allowed us to create major morphogenetic changes in sugarcane (Fig. 3; Supplementary Fig. S6), a key objective of breeding, which could be exploited for increasing sink strength and consequently cane and sugar yield (Fig. 5).

### Differential regulation of sugarcane culm and leaf growth

A strong spatial and organ-specific regulation of GA signaling was observed in sugarcane (Fig. 2). *ScGAI* expression was highest in the shoot apical region and lowest in the mature internodes (Fig. 2). An opposite trend was evident for GA level (Supplementary Fig. S2). This spatial distribution has both structural and functional implications. For instance, previous studies have demonstrated that high cytokinin and low GA levels are required for normal SAM function and plant development (Sakamoto *et al.*, 2001; Jasinski *et al.*, 2005). The gradual basipetal increase in GA content is required to support cell division and expansion in elongating internodes. In addition, the unique nature of transport and accumulation of a large quantity of sucrose involves sucrose breakdown and re-synthesis at the site of storage in sugarcane culms. This is an active process and GA is known to regulate the activity of various enzymes, including hydrolases involved in carbohydrate storage and breakdown (Gibson, 2004; Paparelli *et al.*, 2013). The increasing amount of GA in maturing sugarcane culm may have other roles, as well. For instance, sugarcane culm carries a substantial amount of fibre, of which lignin is a dominant component. A high-carbon environment is needed for lignin biosynthesis and its intensity increases basipetally, paralleling GA content, in developing sugarcane culm. Both GA and sugar regulate lignin production (Biemelt *et al.*, 2004; Huang *et al.*, 2015). Further, sugarcane is a vegetatively propagated plant, and GA breaks bud dormancy and supports plantlet growth by remobilizing carbohydrate reserves (Leduc *et al.*, 2014). So, collectively the longitudinal profile of ScGAI expression and GA activity may be a key determinant of the structural and functional specifications of sugarcane culm.

In contrast to culm, ScGAI in mature leaf tissue was found to be SUMOylated (Fig. 2), indicating that SUMOylation is a dominant regulatory strategy for ScGAI stabilization in sugarcane leaf. Spatially, in monocot leaves, the linear organization comprises dividing cells at the base, followed by expanding cells and finally mature cells at the tip. A local and very narrow peak of GA is present at the transition (i.e. at the base) between the division and expansion zones of maize leaves (Nelissen *et al.*, 2012). In our study, surprisingly, we found evidence that SUMOylation plays an important role in leaf growth through ScGAI stabilization. This strongly supports the idea that, in sugarcane, SUMO-mediated ScGAI stabilization represses the growth of mature leaf tissue.

SUMOylation also plays a central role in environmental responses, such as those to drought and salt stress. In rice and Arabidopsis plants, OTS SUMO proteases are rapidly degraded upon salt stress, leading to an increase in the SUMO conjugation of target proteins (Conti *et al.*, 2008; Srivastava *et al.*, 2016). Previous work has shown that drought stress slows leaf elongation in sugarcane, which reduces photosynthetic area and total plant photosynthesis (Basnayake *et al.*, 2012). In addition, recently, GA biosynthesis was found to be down-regulated in sugarcane leaves under drought stress (Li *et al.*, 2016). These previous reports and our data on SUMOylation of ScGAI led us to speculate that, as in Arabidopsis and rice, OTS protease degradation may contribute to hyper-SUMOylation and stabilization of ScGAI in the elongating and dividing sections of younger sugarcane leaves, upon abiotic stresses, causing rapid cessation of growth.

### Regulation of culm development and modulation of source–sink physiology in sugarcane

The most important agronomic attribute of sugarcane biology is sucrose accumulation in culms, and this is determined by the storage volume capacity (culm volume) and the availability of sucrose for storage. Culm volume is a function of the number and size of the culms and is highly sensitive to environmental conditions, making its improvement by conventional breeding harder. The provision of sucrose for storage is dictated by the demand driven by both growth sinks (root, shoot, and intercalary meristems, and the growing tissues attached to them) and storage sinks (culm volume) and sucrose concentration in culm tissue.

Expanding the culm volume to improve sucrose yield by increasing developmental changes is a logical strategy for sugarcane. This premise is founded on exploiting the principles of source–sink regulation (a supply–demand relationship) and is supported by the following findings. Sugarcane has extraordinary unharnessed photosynthetic plasticity that could be used to increase sugar yield (McCormick *et al.*, 2006), but the realized photosynthetic capacity in sugarcane varieties grown under commercial conditions is much lower, due to sink-limited end-product repression of photosynthesis (Watt *et al.*, 2013). Our findings demonstrated that, while photosynthetic rates were reduced in dwarf ScGAIOE lines, likely due to sink limitation and higher levels of glucose in the leaves (Fig. 5; Supplementary Fig. S10), HpScGAI lines did not show a significant difference in comparison with control plants. This indicates that silencing of sink repressor *ScGAI* might have changed the carbon partitioning and accelerated phytomer production in sugarcane.

In sugarcane, growth inhibitors or ripeners based on hormones, such as Moddus, an inhibitor of GA biosynthesis, or ethephon, an ethylene-releasing compound, are commonly used in the field to enhance sucrose content. In a recent work, we observed that ethephon-treated sugarcane showed a stunted culm and increased *ScGAI* expression level in the upper internodes (Cunha *et al.*, 2017). To extend this further, our findings from *ScGAI*-transgenic sugarcane presented here confirmed the central role of ScGAI in regulating culm growth (Fig. 4). Culm elongation was inhibited and tillering was promoted by GA signaling repression in sugarcane (Fig. 4B; Supplementary Fig. S6). Conversely, the silencing of the *ScGAI* gene resulted in a constitutively active GA response and an earlier onset of internode elongation.

The status of realized commercial cane yield is no different. Average cane yield in most sugarcane-growing countries is less than half of the experimental maximum recorded, even in areas with well-managed pest and disease control, indicating widespread occurrence of growth-limiting environmental conditions (Inman-Bamber, 2013). In a previous study, application of gibberellins increased cane yield by up to 10.9 t ha^−1^ compared with untreated controls in Hawaiian sugarcane production conditions (Moore *et al.*, 1982).

However, the gibberellin response was not always consistent and significant genetic variation was observed in this and related studies. GA and PIFs are essential to promote growth under high carbon availability at night in plants (Stewart *et al.*, 2011). In sugarcane most of the starch turnover (Supplementary Fig. S11) and culm growth occurs at night (Van Dillewijn, 1952). Besides, sucrose was shown to up-regulate PIF1, 3, 4 and 5 levels in the darkness only in the presence of GA (Liu *et al.*, 2011). Based on our results, we infer that ScGAI interacts with ScPIFs, leading to their sequestration and destabilization, and consequently impairing their DNA-binding capacity and thereby blocking PIF interactions with growth-related regulatory genes during the night. Therefore, the protein–protein interactions of ScGAI presented in this study (Fig. 6) indicate that the same mechanism underlying the DELLA action, conserved in other species, is regulating culm growth in sugarcane.

Finally, considering the fact that ScGAI is a single or low copy master regulator of growth with large direct or indirect influence on source–sink regulation, culm growth, and sugar accumulation, it should be one of the prime targets for genetic manipulation by conventional or molecular means, such as gene editing, for variety development.

## Supplementary data

Supplementary data are available at *JXB* online.

Dataset S1. Statistical significance of metabolite variation by Tukey’s test.

Fig. S1. *ScGAI:VENUS* overexpressing transgenic Arabidopsis.

Fig. S2. Gibberellin level along the sugarcane stem.

Fig. S3. Recombinant expression of His-tagged ScGAI.

Fig. S4. PCR genotyping for transgenic sugarcane lines.

Fig. S5. *ScGAI* gene expression transgenic sugarcane lines.

Fig. S6. Gross phenotype transgenic sugarcane plants.

Fig. S7. Leaf and stem histology of transgenic sugarcane plants.

Fig. S8. Gibberellin (GA_3_) and paclobutrazol (PAC) treatments in transgenic sugarcane plants.

Fig. S9. Transcriptional responses of ScGAIOE and HpScGAI plants.

Fig. S10. Photosynthesis in the ScGAIOE and HpScGAI transgenic sugarcane.

Fig. S11. ScGAIOE shows impaired starch accumulation during the day.

Fig. S12. ScPIF3 and 4 proteins in sugarcane.

Fig. S13. ScEIN3 and ScEIL1 in sugarcane.

Table S1. Primers used in this study.

Table S2. Composite list of DEGs in leaves differing between ScGAIOE and HpScGAI.

Table S3. Composite list of DEGs in apical shoot differing between ScGAIOE and HpScGAI.

Table S4. Composite list of DEGs in fifth internode differing between ScGAIOE and HpScGAI.

Table S5. Composite list of DEGs in ninth internode differing between ScGAIOE and HpScGAI.

Supplementary Figures and Tables

Supplementary Dataset S1

## Abbreviations:

DEG: differentially expressed gene
EIL: ethylene insensitive-like
EIN: ethylene insensitive
ERF: ethylene-responsive element binding factor
GA: gibberellin
GA20ox: GA20 oxidase
GID1: GA-insensitive dwarf1
OTS: overly tolerant to salt1
PAC: paclobutrazol
PIF: phytochrome-interacting factor
SIM: SUMO-interacting motif
SUMO: small ubiquitin-like modifier.
