# Supplementary material for: ScGAI is a key regulator of culm development in sugarcane
Source: J Exp Bot. 2018 May 21;69(16):3823–37. doi: 10.1093/jxb/ery180 (PMC6054169; doi:10.1093/jxb/ery180)
Supplement: Supplementary Figures and Tables [file ery180_suppl_supplementary_figures_and_tables.docx]

**Supplementary data for**

**ScGAI is a key regulator of culm development in sugarcane**

Rafael Garcia Tavares, Prakash Lakshmanan, Edgar Peiter, Anthony O’Connell, Camila Caldana, Renato Vicentini, José Sérgio Soares, Marcelo Menossi*

correspondence to: [menossi@lgf.ib.unicamp.br](mailto:menossi@lgf.ib.unicamp.br)

**
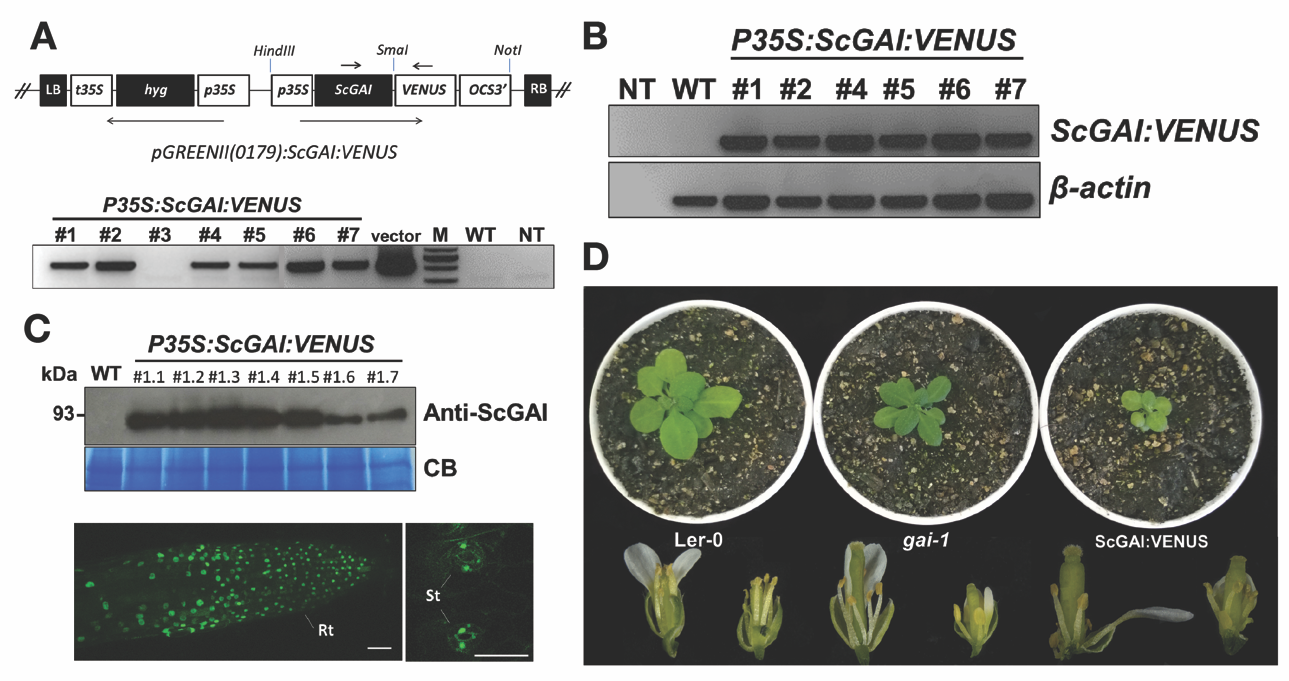
**

**Fig. S1. ScGAI:VENUS fusion protein repressed the GA-responses in transgenic Arabidopsis.**

**A.** Schematic representation of the pGREENII(179):ScGAI:VENUS fusion construct. LB and RB: left and right borders on the T-DNA, respectively; P35S: 35S promoter; T35S: 35S terminator; OCS3’: octopine synthase terminator; Hyg: hygromycin resistance gene. Below, *ScGAI* transgene integration in transgenic plants using specific primers as indicated with an arrow in the diagram. **B.** RT-PCR analysis of *35S:ScGAI:VENUS* expression in transgenic plants. The β-actin gene was used as a loading control. WT: wild type; NT: non-template. **C.** Above, immunoblot analysis of ScGAI:VENUS fusion protein from T2 generation . WT: wild type; CB: Commassie blue-stained membrane as loading control. Below, nuclear fluorescent signal of ScGAI:VENUS fusion protein in root tip (Rt) and stomata (St) visualized by confocal laser microscopy. Bars = 50 µm. **D.** 2-week-old ScGAI:VENUS transgenic Arabidopsis showing a dwarf phenotype in comparison with wild-type (Ler-0) and gibberellin-insensitive (*gai-1*) mutant, and floral buds with short stamen filaments in *gai-1* and ScGAI:VENUS plants.


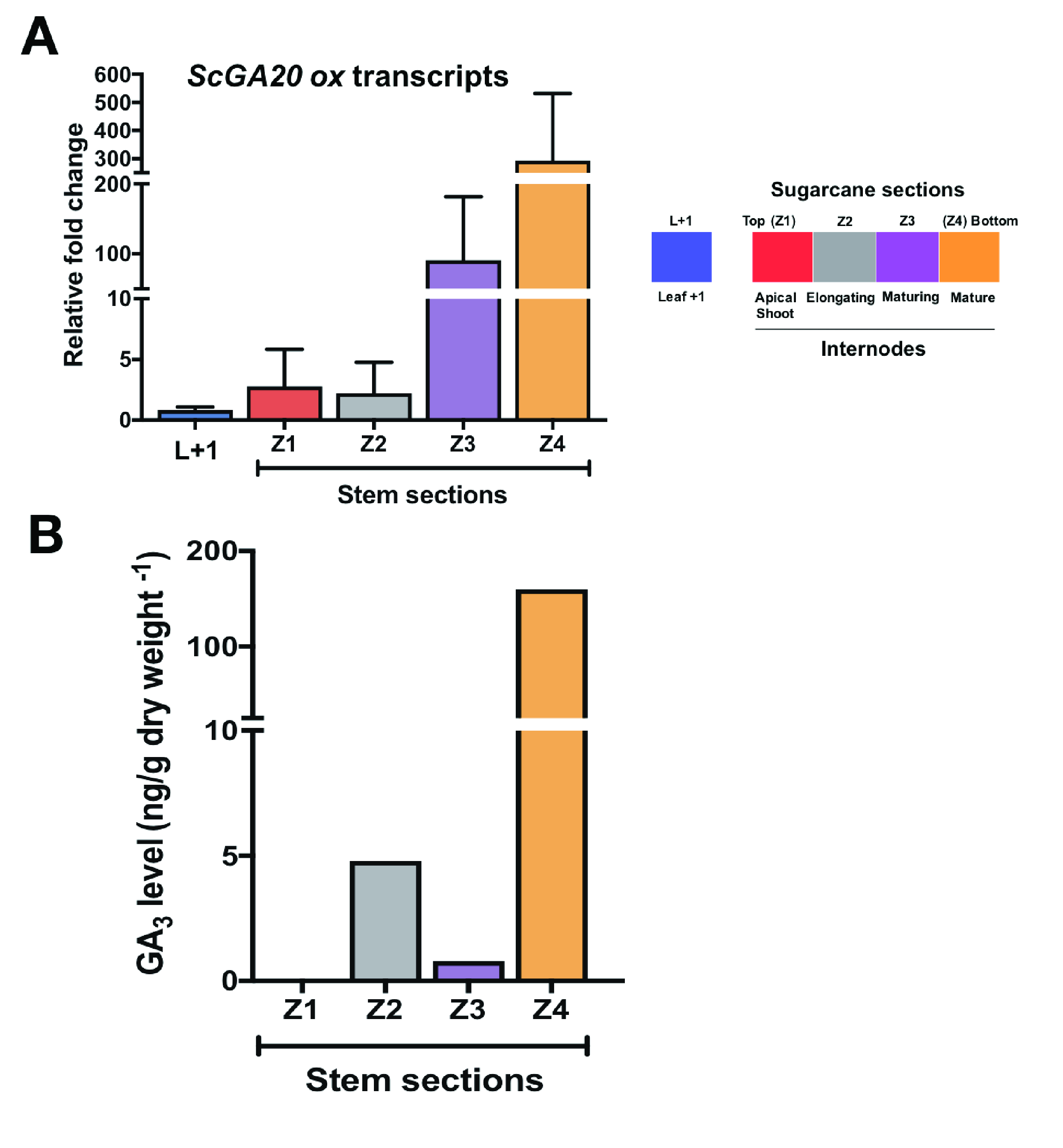


Fig. S2. GA hormone is synthesized in the basal internodes in sugarcane.

A. Expression profile of the native *ScGA20* oxidase gene in different tissues of 10-month-old sugarcane; Bar plots show means ±SD of five biological replicates. B. GA_3_ hormone level in sugarcane tissues. Neither GA_1_ nor GA_4_ were detected in the analyzed samples. Each samples correspond to a pool from five biological replicates.


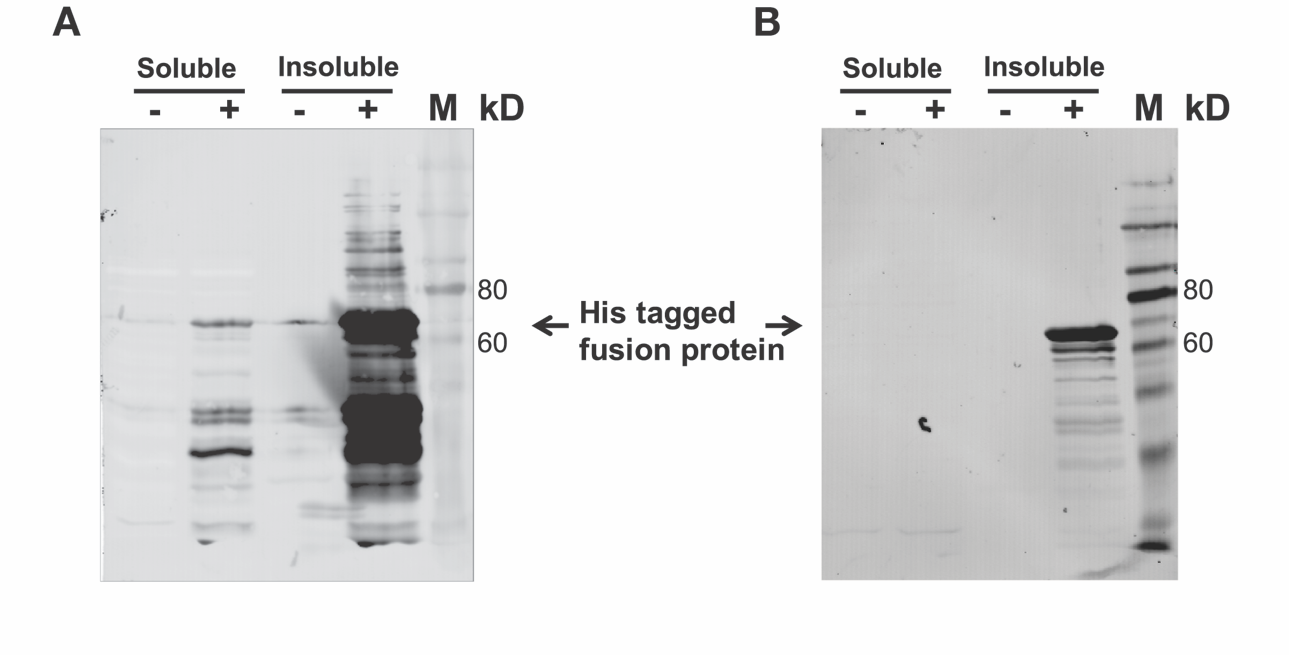


Fig. S3. Expression of His-tagged ScGAI protein conferred its molecular weight of 66 kDa in *Escherichia coli* (*E.coli*).

Expression of recombinant ScGAI protein was induced in *E. coli*. Total protein was extracted and assessed by immunoblotting using the **A.** anti-ScGAI (1:1000 dilution) and **B.** the anti-His (1:1000 dilution) antibodies; lane 1 and 3, non-induced extracts, and lane 2 and 4, induced extracts. Lane 5, Molecular weight marker; Recombinant proteins are indicated with arrows.


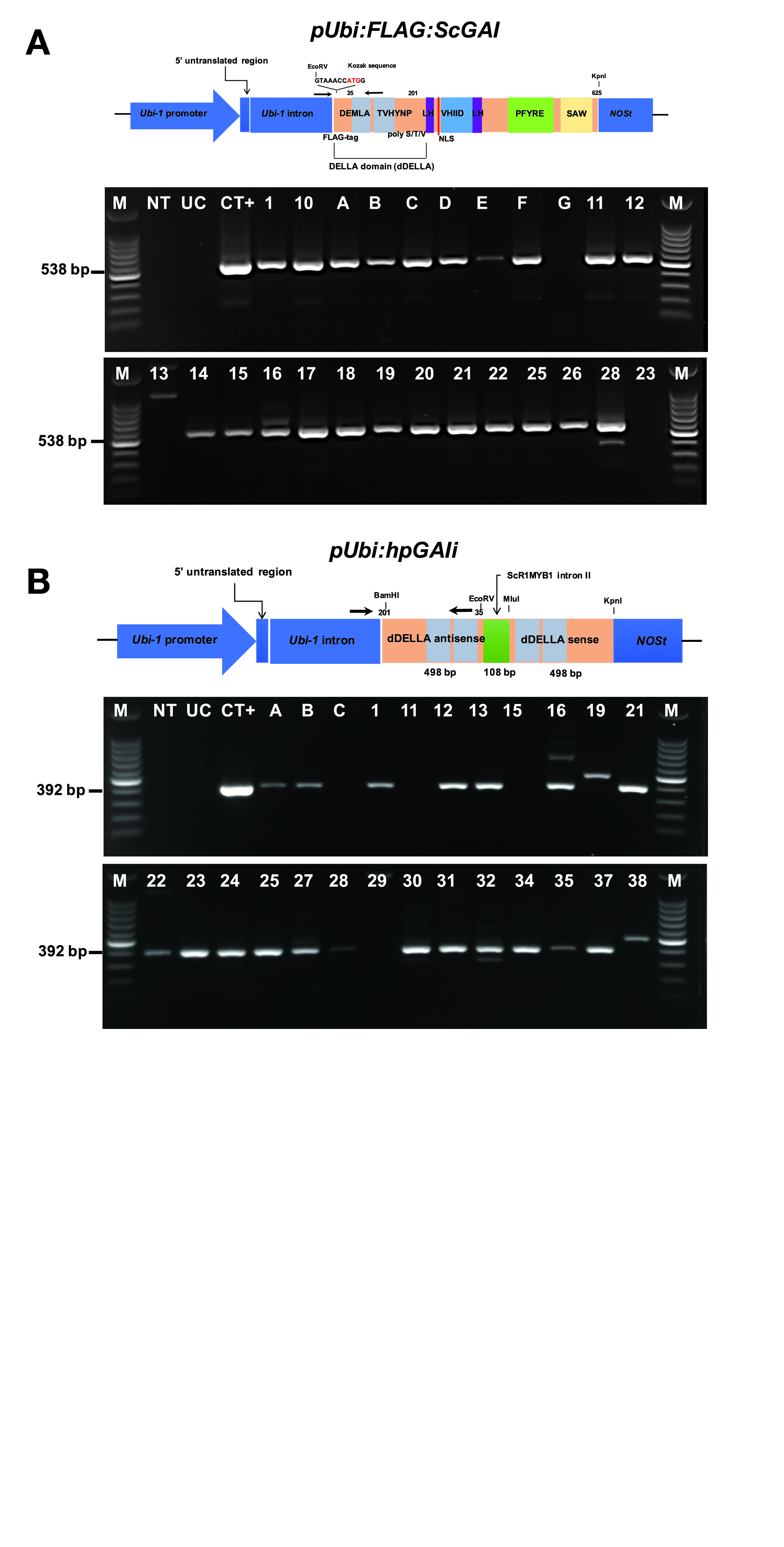


Fig. S4. Transgene constructs and PCR genotyping for identification of putative transgenic lines.

**A.** Overexpression cassette pUbi:FLAG:ScGAI and the positive transgenic lines identified with the expected PCR band. **B.** Hairpin-mediated silencing cassette pUbi:hpGAIi and the positive lines confirmed by the expected PCR band. M: 100 bp ladder (Promega); NT: non-template; UC: untransformed control; CT+: positive control (vectors); Numbers and letters correspond to the putative transformed lines; The set of primers used is shown as arrows in each construct diagram.


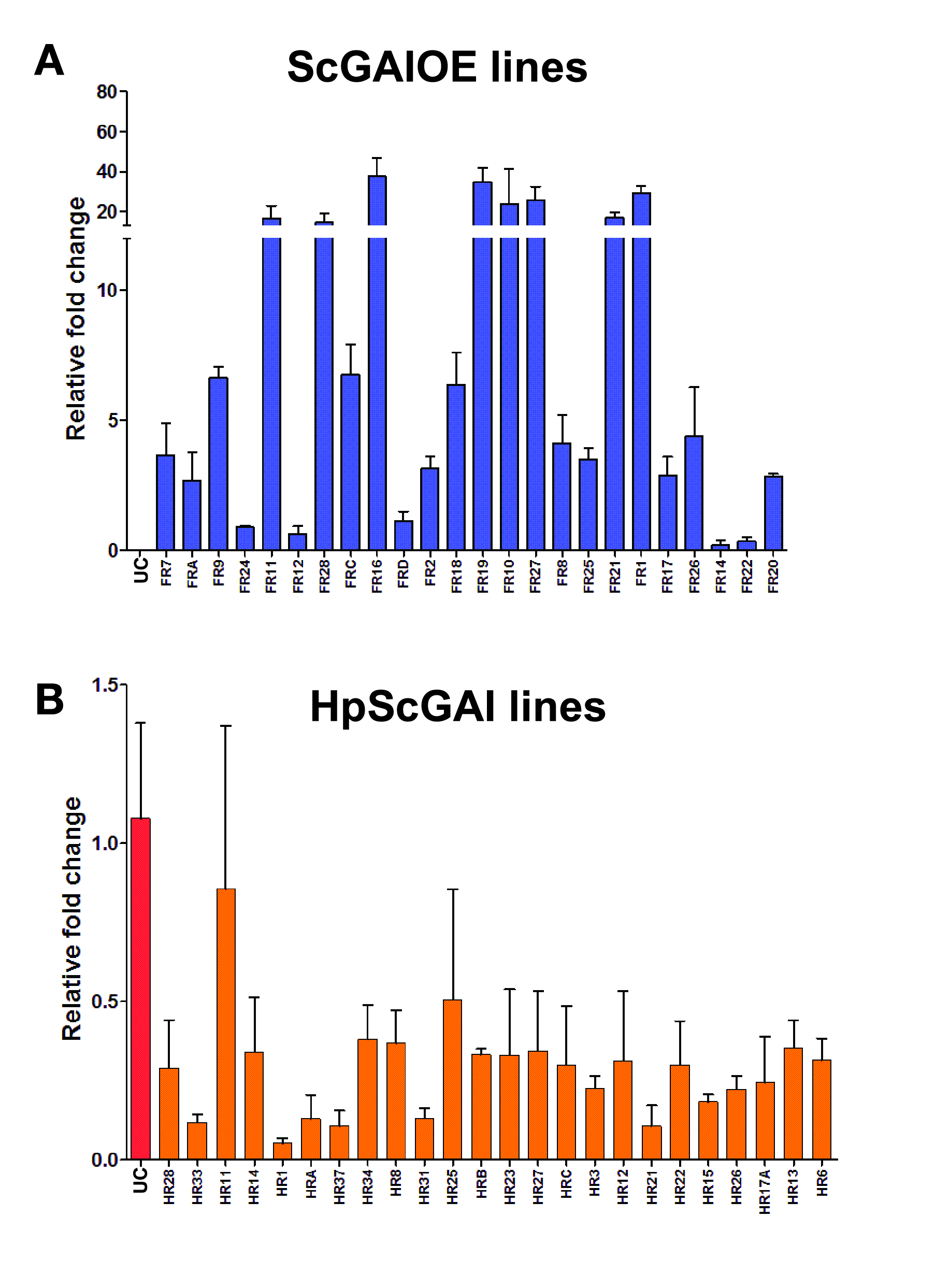


Fig. S5.  *ScGAI* gene expression in the ScGAIOE and HpScGAI transgenic sugarcane lines.

**A.** Expression level of the *FLAG:ScGAI* transgene. **B.** Expression level of endogenous *ScGAI* in untransformed control (UC) and hairpin-mediated DELLA silencing lines. Data represent the mean ±SD of three independent biological replicates.


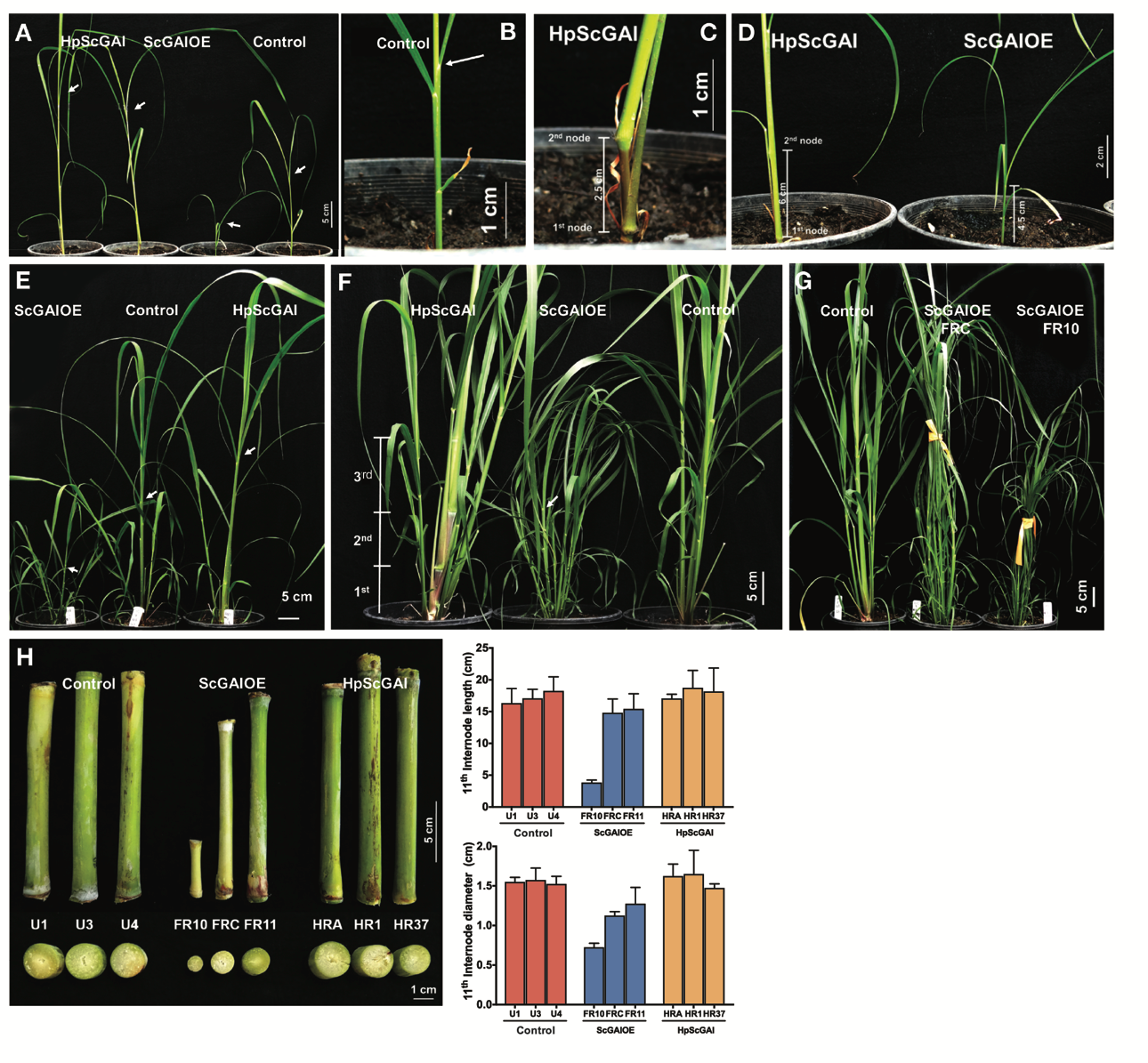


Fig. S6. Gross phenotype of ScGAIOE and HpScGAI transgenic sugarcane plants.

1-month-old in **A** and close-up in (**B, C** and **D**) showing the earliest onset of internodes in HpGAI lines; **E.** 2-month-old; **F.** and **G** 3-month-old showing the high tiller numbers. Arrows indicate the first visible dewlap; **H.** 11^th^ internode length and diameter measurement of 6-month-old plants. Bar plots show means ±SD of four biological replicates. U1, U3 and U4: untransformed controls; FR10, FRC and FR11: ScGAIOE plants; HRA, HR1 and HR37: HpScGAI plants.


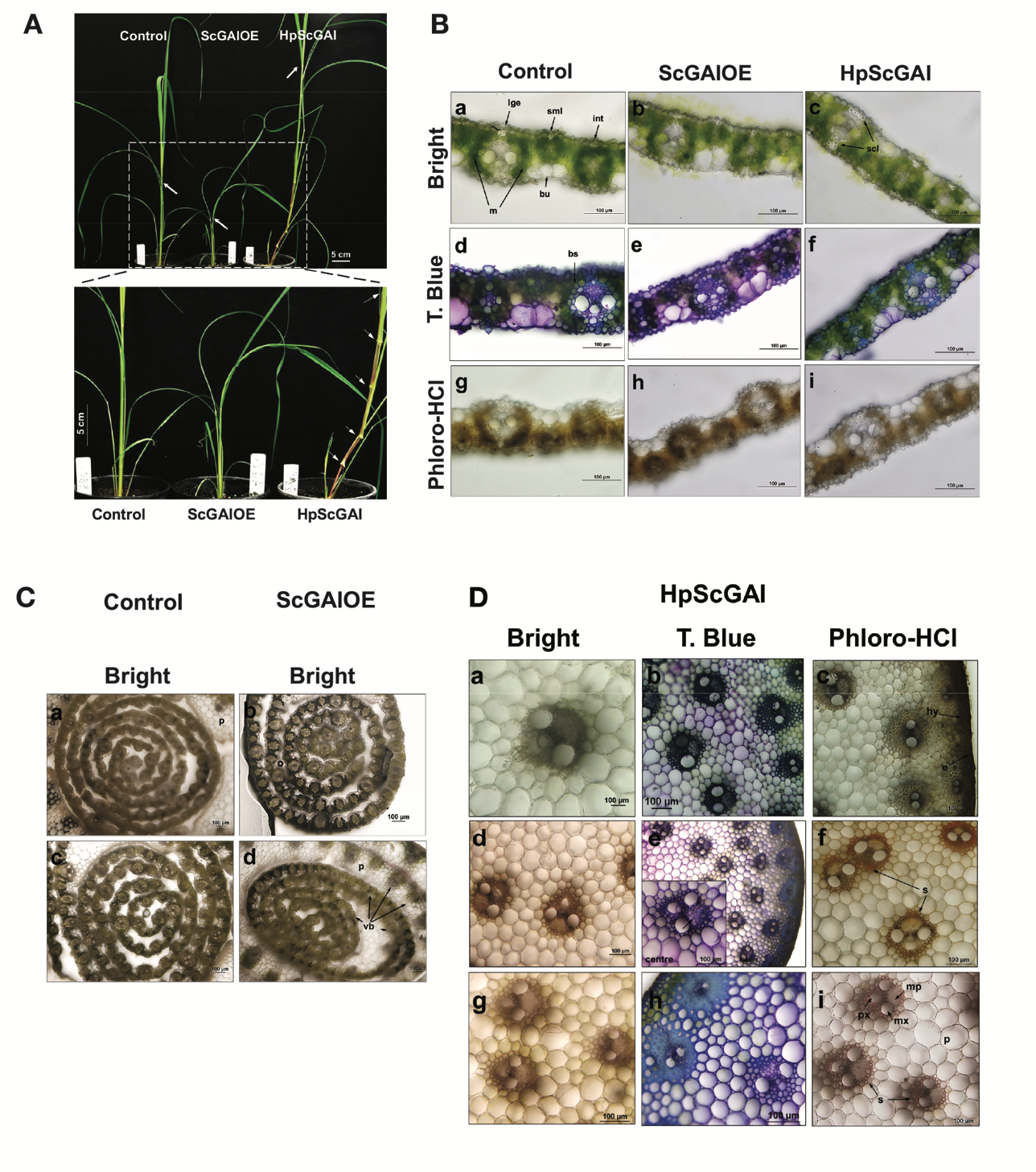


Fig. S7. Leaf and stem histology of control, ScGAIOE and HpScGAI transgenic sugarcane plants.

**A.** 3-month-old plants. Close-up view displaying the stunted growth in ScGAIOE and the presence of internodes in HpScGAI plants. Arrows indicate the first visible dewlap; **B.** Cross section of leaves in bright-field light **a**,**b** and **c**, and stained with toluidine blue (T.blue) **d**, **e** and **f** or phloroglucinol-HCl (Phloro-HCl) **g**, **h** and **i**. Large (lge), small (sml) and intermediate (int) vascular bundle, scl – sclerenchyma, bu – buliform cells, bs – bundle sheath, m- mesophyll cell; **C.** Cross-section of the leaf roll from control in bright-field in **a** and **c**, and from ScGAIOE (FR10 line) in bright-field in **b** and **d**; **D.** Cross-section of the stem from HpScGAI plants (HR1 line) in bright-field light in **a**, **d** and **g**, toluidine blue (T.blue) stained in **b**, **e** and **h**, and phloroglucinol-HCl (Phloro-HCl) stained in **c**, **f** and **i**. Parenchyma cells (p), vascular bundles (vb), metaxylem (mx), protoxylem (px), metaphloem (mp), epidermis (e), sclerenchyma (ex), hypodermis (hy).


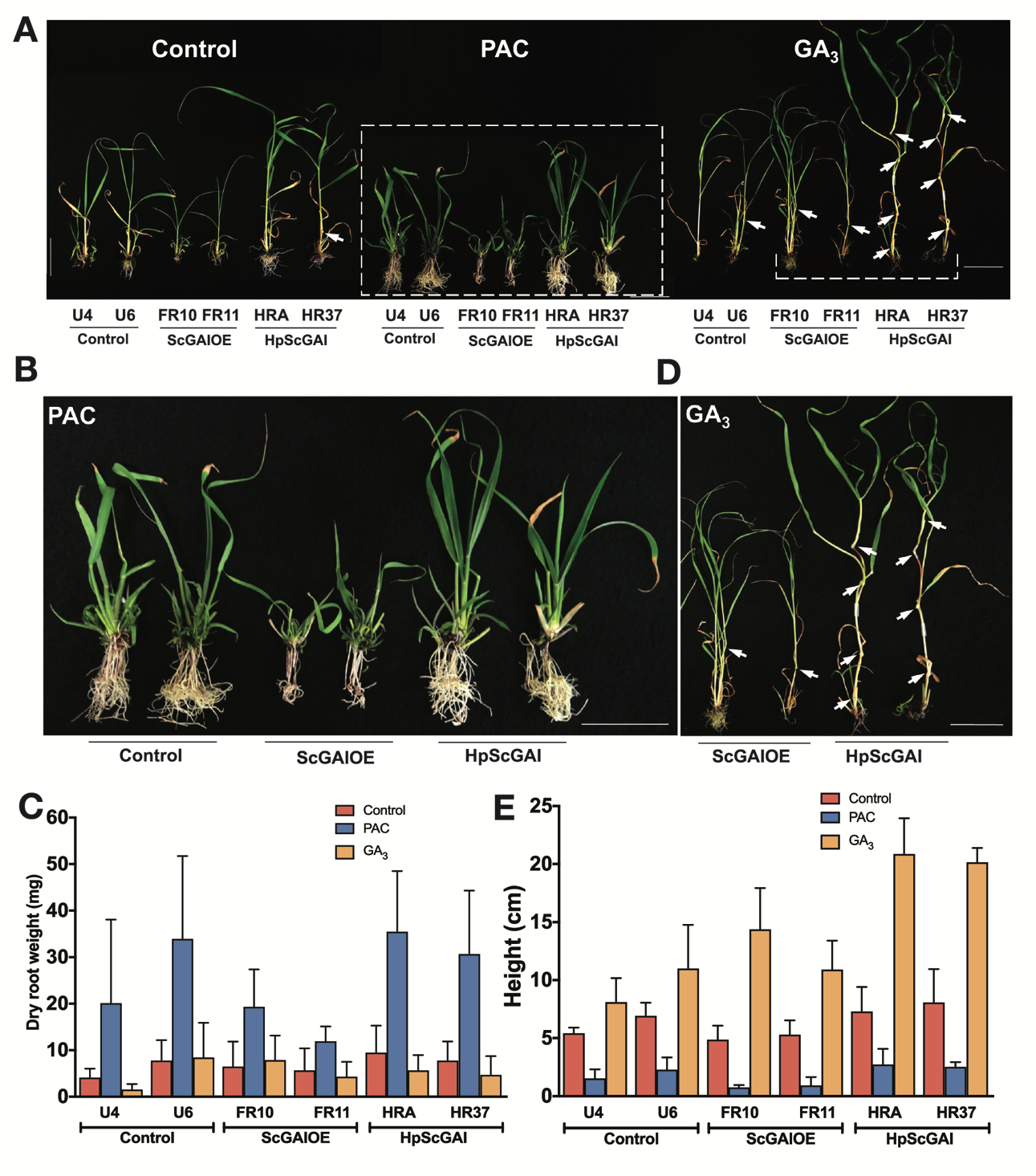


Fig. S8. Transgenic sugarcane plants showed stronger responses to gibberellin (GA_3_) and paclobutrazol (PAC) than control plants.

**A.** Morphology of sugarcane plants after 23 days of treatment with GA_3_ **(**50µM) and PAC (5 µM). Arrows indicate the nodes. Scale bars = 5 cm. **B.** Close-up view of PAC-treated seedlings. Scale bars = 5 cm. **C.** Dry root weight of control, ScGAIOE and HpScGAI plants. **D.** Close-up view of GA_3_-treated transgenic plants. Arrows indicate the nodes; Scale bars = 5 cm. **E.** Height of control, ScGAIOE and HpScGAI transgenic lines. Error bars indicate the SD of the mean of four biological replicates.

**
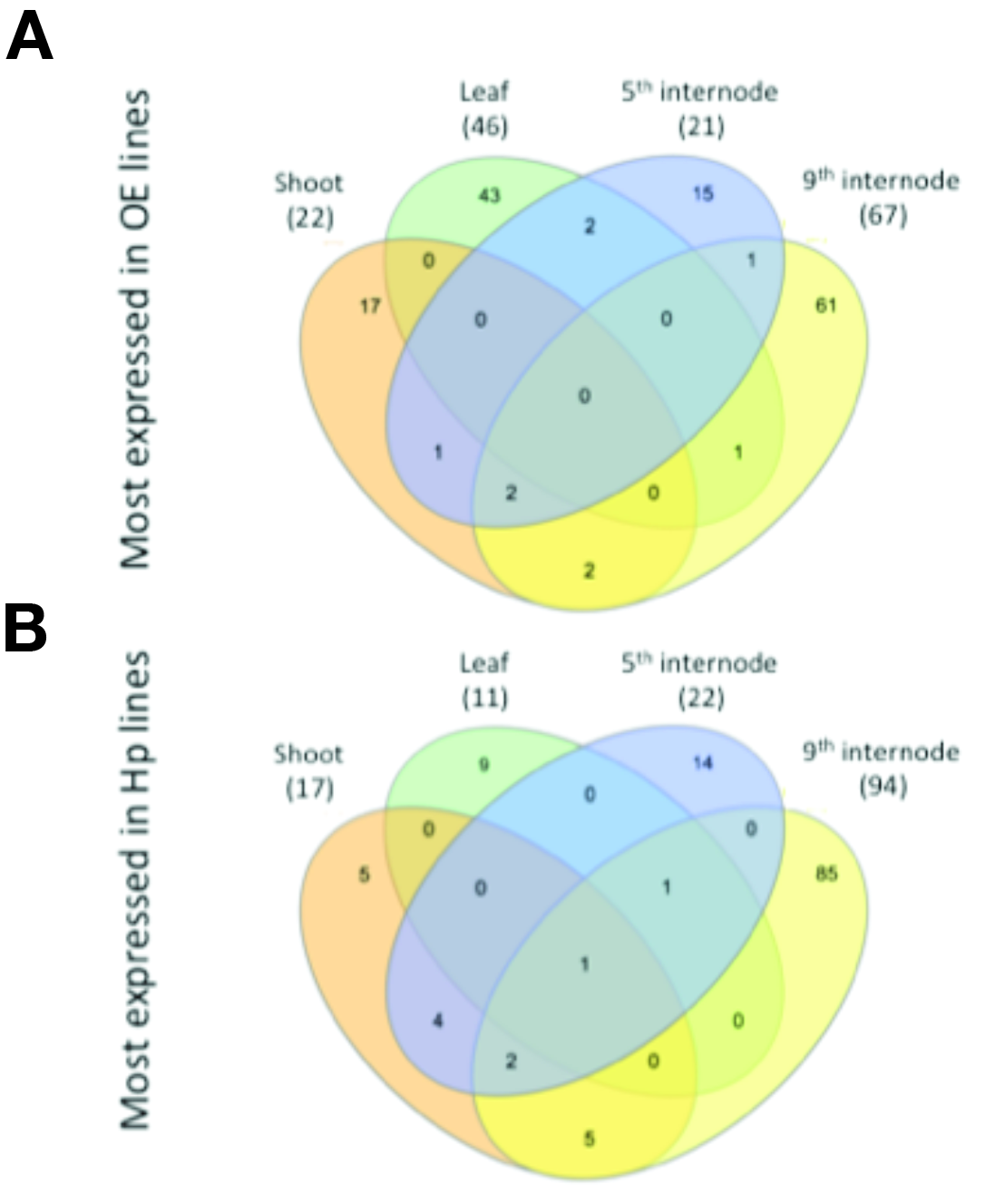
**

Fig. S9. Transcriptional responses of ScGAIOE and HpScGAI plants.

Venn diagram showing the differentially expression genes (DEGs) (number in parentheses) **A.** in ScGAIOE and **B.** in HpScGAI tissues.


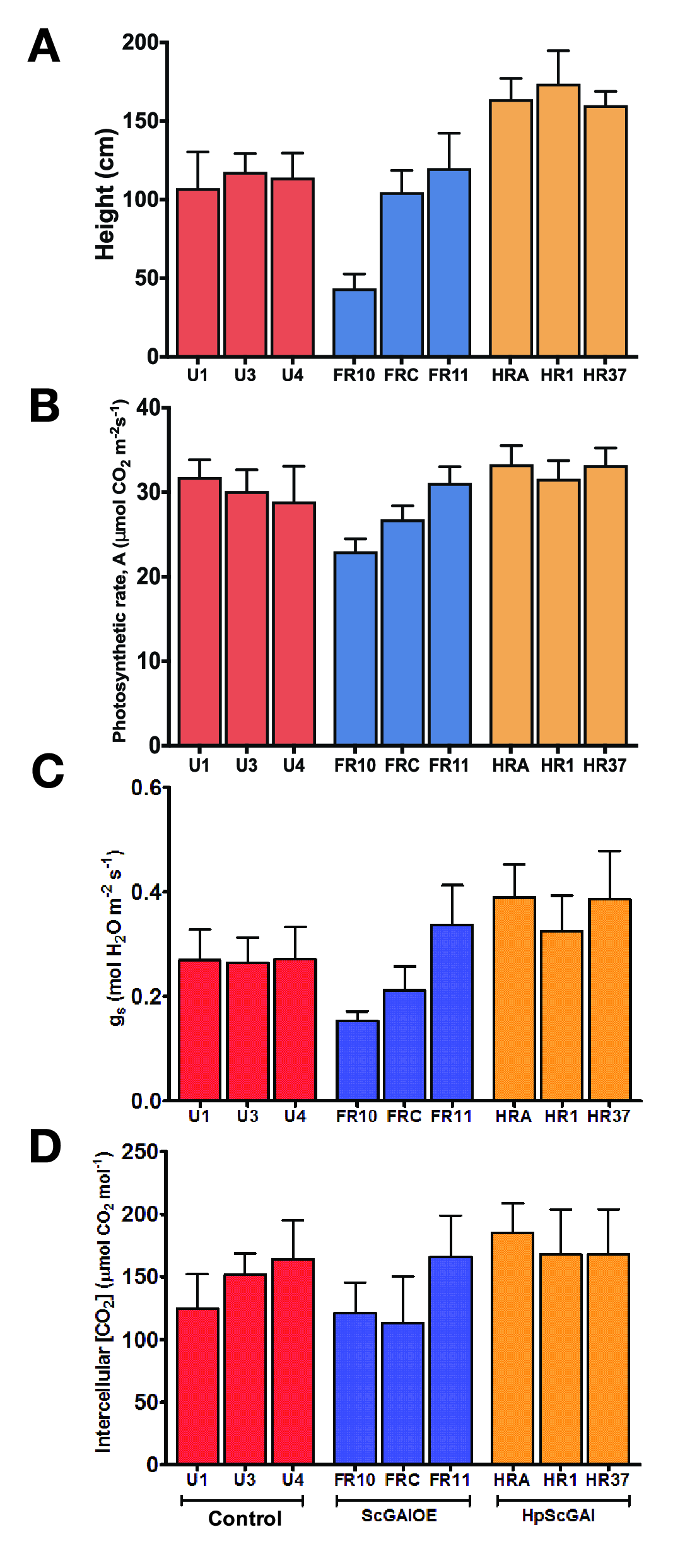


Fig. S10. Photosynthesis in the ScGAIOE and HpScGAI transgenic sugarcane.

**A.** Height, **B.** Photosynthesis rate A., **C.** Stomatal conductance (*gs*) and **D.** Intercellular [CO_2_], (*c_i_*). Parameters were measured in 5-month-old plants. Bar plots show means ±SD of seven biological replicates.


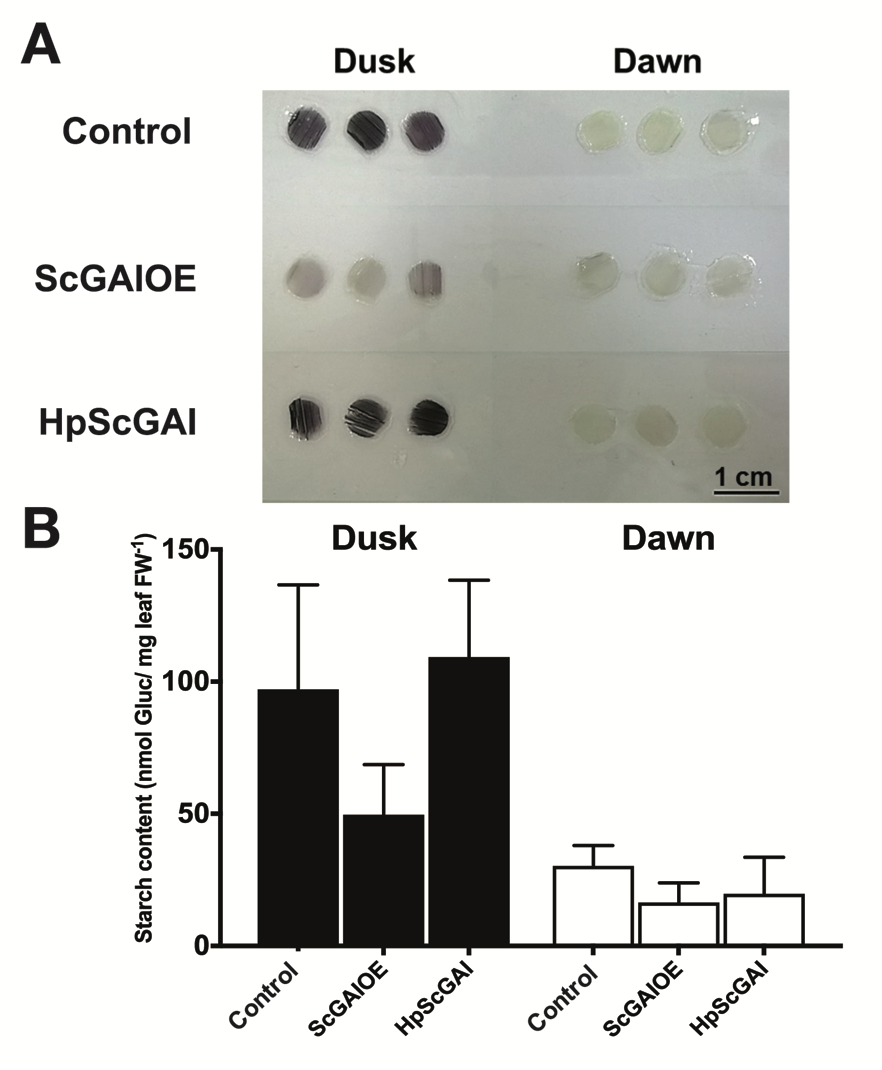


**Fig. S11.** **ScGAIOE shows impaired starch accumulation during the day.**

Starch turnover was evaluated in diurnal cycle by **A.** Lugol’s iodine solution in leaf+1 discs of three independent biological replicates and confirmed by **B.** enzymatic starch assay of five independent biological replicates.


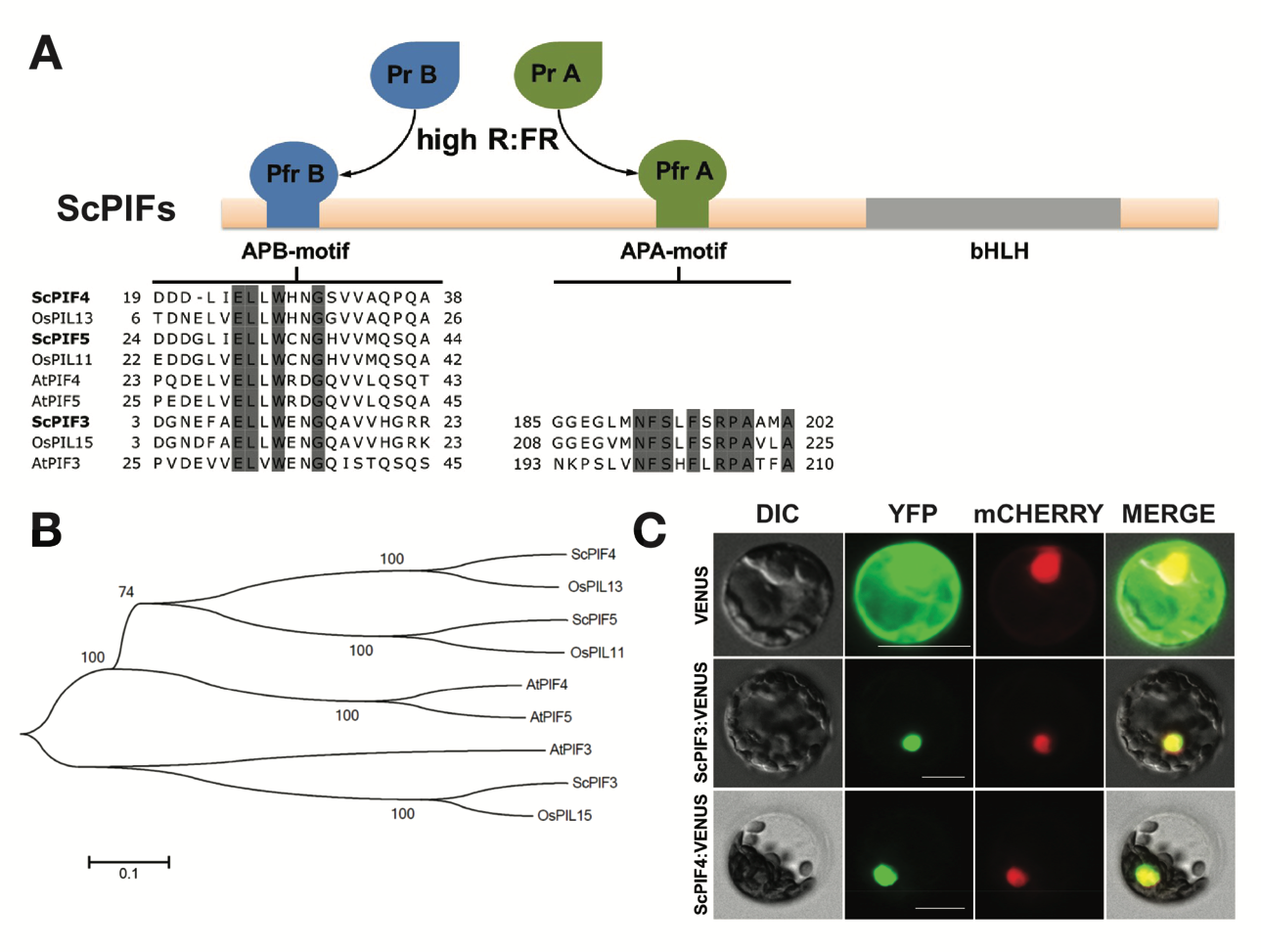


Fig. S12. Phytochrome-Interacting Factors (PIFs) 3 and 4 are nuclear basic helix-loop-helix (bHLH) proteins in sugarcane.

**A.** Schematic representation of PIF proteins showing their conserved domains along the sequence. APB and APA-motifs mediate the binding to phyB Pfr and phyA Pfr, respectively; **B.** Phylogenetic tree of PIF proteins. **C.** Subcellular localization of ScPIF:VENUS fusion proteins in Arabidopsis mesophyll protoplast. The construct *AtPARP3:mCHERRY* (mCHERRY) was used as nuclear control. DIC: Differential Interference Contrast; YFP: Yellow Fluorescent protein. Bars = 20 µM


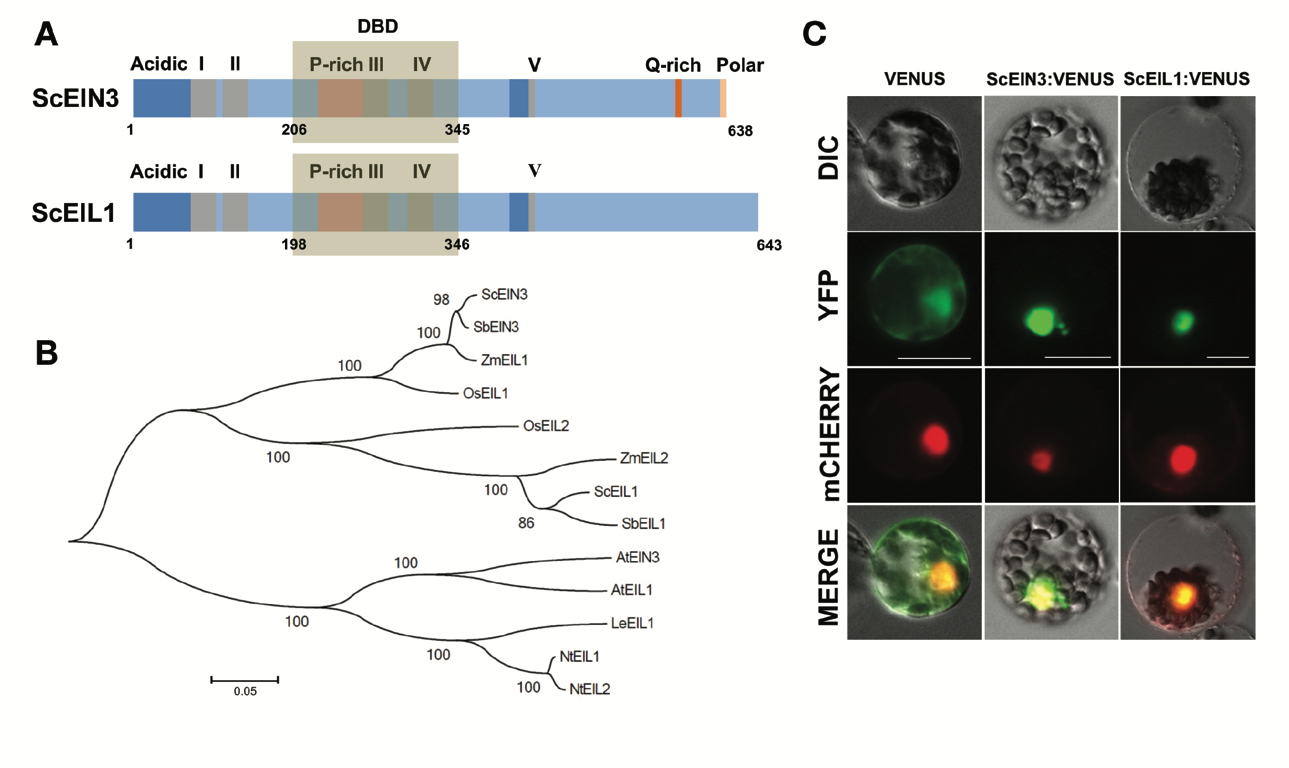


Fig. S13. ScEIN3 and ScEIL1, the master transcription factors of ethylene signaling.

**A.** Schematic representation of ScEIN3/EIL1 proteins showing their conserved domains along the sequences. DBD means DNA binding domain. P-rich means proline rich and Q-rich means glutamine rich domains. The numbers are indicating the five basic domains; **B.** Phylogenetic tree of ScEIN3/EIL1 proteins. **C.** Subcellular localization of ScEIN3/EIL1 proteins in Arabidopsis mesophyll protoplast. The construct *AtPARP3:mCHERRY* (mCHERRY) was used as nuclear control. DIC means Differential Interference Contrast. YFP means Yellow Fluorescent protein. Bars = 20 µM

**Table S1.** Composite list of DEGs in leaves between ScGAIOE and HpScGAI.

| **GeneID** | **Annotation** | **ScGAIOE** | **HpScGAI** | **Fold change (Log2)** | **q-values** |
| --- | --- | --- | --- | --- | --- |
| Sb01g007360 | Gibberellic acid -stimulated Arabidopsis (GASA8) gene | 3.63978 | 0 | inf | 0.018663 |
| Sb01g029310 | Expansin B2 – (EXPB2) | 1.82334 | 0 | inf | 0.018663 |
| Sb01g036580 | Euonymus lectin S3 - EULS3 | 125.358 | 12.1324 | -3.36912 | 0.018663 |
| Sb01g042690 | Sugar transporter family protein – (STP13) | 43.3993 | 7.30469 | -2.57078 | 0.032348 |
| Sb01g043720 | Glycosyl hydrolase family 10 protein | 0.990486 | 0 | inf | 0.018663 |
| Sb01g044580 | Alcohol dehydrogenase GroES-like | 68.0263 | 11.0143 | -2.62672 | 0.04479 |
| Sb01g007590 | UDP-glucuronosyl/UDP-glucosyltransferase | 0 | 3.645 | inf | 0.032348 |
| Sb01g010660 | DELLA | 39.7408 | 4.80204 | -3.0489 | 0.018663 |
| Sb01g020150 | KAT2 – 3- Ketoacyl-Coa thiolase 2 | 252.081 | 23.083 | -3.44899 | 0.018663 |
| Sb01g033820 | Transducin/WD40 repeat-like superfamily protein | 57.6083 | 7.64138 | -2.91437 | 0.018663 |
| Sb01g042270 | Cytochrome P450 74A – (CYP74A) | 360.304 | 52.1615 | -2.78816 | 0.032348 |
| - | NI | 3.43827 | 0 | inf | 0.018663 |
| Sb10g029300 | Thylakoid lumenal 16.5 kDa protein | 23.6458 | 123.636 | 2.38644 | 0.04479 |
| Sb10g028360 | GDSL-like Lipase/Acylhydrolase | 15.4885 | 82.5155 | 2.41347 | 0.04479 |
| Sb02g037570 | Glucose transmembrane transporter - Polyol transporter 5-like | 18.154 | 1.98172 | -3.19546 | 0.032348 |
| Sb02g037650 | Scarecrow-like 5 | 216.577 | 18.8808 | -3.51989 | 0.018663 |
| Sb02g003010 | Early-responsive to dehydration – (ERD4) | 182.848 | 5.04105 | -5.18078 | 0.018663 |
| Sb02g007870 | Metal transporter Nramp6 – (NRAMP1) | 1.79574 | 0 | inf | 0.018663 |
| Sb02g010810 | Aquaporin-like – (PIP2B) | 0 | 2.58132 | inf | 0.018663 |
| Sb02g026360 | Galactosyltransferase | 33.9953 | 2.60119 | -3.70809 | 0.018663 |
| Sb02g031550 | Copper amine oxidase | 0 | 2.81452 | inf | 0.018663 |
| Sb02g036750 | Polygalacturonase inhibitor 1 – (PGIP2) | 2.29455 | 0 | inf | 0.018663 |
| - | NI | 0 | 51.842 | inf | 0.018663 |
| Sb03g000850 | Putative bark storage protein | 104.951 | 17.2066 | -2.60868 | 0.018663 |
| Sb03g029790 | CTP synthase – (emb2742) | 40.2019 | 5.45801 | -2.88082 | 0.018663 |
| Sb03g007380 | Mannose-6-phosphate isomerase – (PMI1) | 9.95695 | 56.4115 | 2.50221 | 0.018663 |
| Sb03g023990 | Early-responsive to dehydration – (ERD) | 76.1976 | 11.114 | -2.77737 | 0.018663 |
| Sb03g030330 | MYB family transcription factor – (RL6) | 0 | 46.0747 | inf | 0.018663 |
| Sb03g040490 | C2H2 zinc finger protein – (WIP4) | 0.803423 | 0 | inf | 0.018663 |
| Sb03g042450 | Lipoxygenase – (LOX1) | 318.627 | 28.708 | -3.47234 | 0.018663 |
| Sb03g044980 | Glutathione S-transferase – (GSTF13) | 109.402 | 21.297 | -2.36092 | 0.018663 |
| Sb03g046090 | BHLH039 | 3.72414 | 0 | inf | 0.018663 |
| Sb04g000620 | Vacuolar invertase 2 – (VAC-INV 2) | 83.5824 | 14.485 | -2.52864 | 0.032348 |

“NI” means no identified; “inf” indicates no ratio.

**Table S1**. Continuation.

| **GeneID** | **Annotation** | **ScGAIOE** | **HpScGAI** | **Fold change (Log2)** | **q-values** |
| --- | --- | --- | --- | --- | --- |
| Sb04g000830 | HPL1 (Hydroperoxide Lyase 1) (CYP74B2) | 36.3315 | 3.85201 | -3.23754 | 0.018663 |
| Sb04g021410 | Early-responsive to dehydration – (ERD1) | 92.2606 | 14.9928 | -2.62145 | 0.018663 |
| Sb04g024090 | NPF8.3. NRT1/ PTR family 8.3 - Peptide transporter (PTR2) | 118.224 | 13.927 | -3.08557 | 0.018663 |
| Sb04g024440 | Glycerophosphoryl diester phosphodiesterase – (GPDL2) | 27.8855 | 3.19227 | -3.12686 | 0.018663 |
| Sb04g033350 | Cytochrome b5-like Heme/Steroid binding domain -CB5-E | 8.58836 | 0 | inf | 0.018663 |
| Sb04g025550 | APK1 - Serine-threonine/tyrosine-protein kinase | 246.684 | 28.4504 | -3.11615 | 0.018663 |
| Sb04g026690 | Uncharacterized protein | 4.67203 | 0 | inf | 0.018663 |
| Sb04g031040 | Jumonji transcription factor/ zinc finger (C5HC2 type) | 23.0275 | 1.20107 | -4.26097 | 0.018663 |
| - | NI | 0 | 20.0573 | inf | 0.018663 |
| - | NI | 48.5983 | 319.451 | 2.71662 | 0.018663 |
| Sb05g003860 | LTP1 – Lipid transfer protein 1 | 4.46598 | 0 | inf | 0.018663 |
| - | NI | 0 | 4.64044 | inf | 0.018663 |
| - | NI | 0 | 3.87019 | inf | 0.018663 |
| Sb06g021790 | Wall-associated receptor kinase-like 20 precursor-  (CRCK3) | 74.8249 | 14.0611 | -2.41181 | 0.04479 |
| Sb06g028200 | Protease inhibitor/seed storage/LTP family | 243.106 | 34.7061 | -2.80832 | 0.018663 |
| Sb06g000660 | Heat shock protein – (HSP90.1) | 9.17577 | 60.5537 | 2.72231 | 0.018663 |
| Sb06g003280 | HIPP27 - Heavy metal associated isoprenylated plant protein 27 | 112.18 | 8.56149 | -3.71181 | 0.018663 |
| Sb06g022460 | Beta-glucosidase 45 – (BGLU45) | 15.1642 | 77.432 | 2.35226 | 0.032348 |
| Sb06g027770 | ACA8 (auto-inhibited CA^2+^ -ATPASE. isoform 8) | 19.1969 | 2.89684 | -2.72832 | 0.04479 |
| Sb06g032460 | PAO4. Polyamine Oxidase 4 | 64.6633 | 10.8507 | -2.57516 | 0.018663 |
| Sb07g004700 | Chalcone synthase – (CHS) transparent testa 4. TT4 | 0 | 2.47114 | inf | 0.018663 |
| Sb07g005130 | Terpene synthase – (TPS21) | 183.727 | 21.3089 | -3.10804 | 0.018663 |
| Sb07g021950 | Receptor-like protein kinase precursor – (PEPR1) | 46.2865 | 3.24164 | -3.83579 | 0.018663 |
| Sb07g024030 | Oxidoreductase. 2OG-Fe(II) oxygenase family protein | 1.12088 | 0 | inf | 0.018663 |
| Sb08g023140 | AAA-ATPASE 1 | 36.0458 | 3.73987 | -3.26877 | 0.032348 |
| Sb08g023150 | AAA-ATPASE 1 | 46.4186 | 5.42508 | -3.09699 | 0.018663 |
| - | NI | 0 | 15.3463 | inf | 0.018663 |
| Sb09g001020 | PR (pathogenesis-related) -PR-6 proteinase inhibitor family | 2615.37 | 278.046 | -3.23362 | 0.018663 |
| Sb09g001050 | PR (pathogenesis-related) -PR-6 proteinase inhibitor family | 1802.67 | 189.723 | -3.24817 | 0.018663 |
| Sb09g003060 | Proteolipid membrane potential modulator – (RCI2A) | 499.02 | 47.4797 | -3.39371 | 0.018663 |
| Sb09g005800 | Histidine-containing phosphotransfer protein 4 – (AHP4) | 0 | 5.34066 | inf | 0.018663 |
| - | NI | 0 | 80.8544 | inf | 0.032348 |

“NI” means no identified; “inf” indicates no ratio.

**Table S2.** Composite list of DEGs in Apical shoot between ScGAIOE and HpScGAI.

| **GeneID** | **Annotation** | **ScGAIOE** | **HpScGAI** | **Fold change (Log2)** | **q-values** |
| --- | --- | --- | --- | --- | --- |
| Sb01g016810 | DYL1 (Dormancy-associated protein-like 1) | 109,977 | 33,15 | -1,73013 | 0,0264797 |
| Sb01g028256 | Tetratricopeptide repeat 10, TPR10 | 0 | 3,05063 | inf | 0,0264797 |
| Sb01g029610 | LTPL144 - Protease inhibitor/seed storage/LTP family protein precursor | 4,14948 | 0 | #NOME? | 0,0477927 |
| Sb01g036310 | HAD superfamily phosphatase | 135,969 | 26,6852 | -2,34917 | 0,0264797 |
| Sb01g045720 | sucrose transporter, SUC3 | 40,2308 | 10,813 | -1,89553 | 0,0264797 |
| Sb01g008350 | transmembrane BAX inhibitor motif-containing protein BIL4 | 43,0404 | 194,978 | 2,17955 | 0,0264797 |
| Sb01g014460 | VRN1 –Reduced vernalization response 1 | 2,80241 | 0 | #NOME? | 0,0264797 |
| - | NI | 0 | 18,0663 | inf | 0,0477927 |
| - | NI | 0 | 4,02684 | inf | 0,0477927 |
| Sb10g003890 | GDSL-like lipase/acylhydrolase | 143,286 | 42,2648 | -1,76137 | 0,0264797 |
| Sb10g012970 | Peptidyl-prolyl cis-trans isomerase FKBP65 – ROF2 | 50,2692 | 1091,04 | 4,43988 | 0,0264797 |
| Sb10g019360 | AQP1 –Delta tonoplast integral protein | 89,4797 | 6,54048 | -3,77409 | 0,0264797 |
| Sb10g026090 | Chloride transporter A – CLC-A | 16,1588 | 3,30871 | -2,28798 | 0,0264797 |
| Sb02g027900 | Photosystem I subunit G - PSAG | 250,123 | 91,8949 | -1,44458 | 0,0264797 |
| Sb02g032040 | Chlorophyll A-B binding protein LHB1B2,LHCB1.5 | 134,08 | 47,9085 | -1,48474 | 0,0264797 |
| Sb02g035600 | Beta-amylase 1 – RAM1 | 66,8796 | 223,019 | 1,73753 | 0,0264797 |
| Sb02g043260 | Euonymus lectin S3 - EULS3 | 148,289 | 43,8698 | -1,75711 | 0,0264797 |
| Sb03g005280 | FLA11 - Fasciclin-like arabinogalactan proteins | 242,591 | 86,7392 | -1,48377 | 0,0264797 |
| Sb03g006880 | HSP18.2 – Heat shock | 134,315 | 397,858 | 1,56663 | 0,0264797 |
| Sb03g041190 | NPF5.10 – Peptide transporter (PTR2) | 50,1346 | 15,1805 | -1,72359 | 0,0264797 |
| Sb03g006870 | HSP18.2 – Heat shock | 227,072 | 789,49 | 1,79777 | 0,0264797 |
| Sb03g042330 | MLP423- Pathogenesis-related Bet v I family protein | 3,32405 | 0 | #NOME? | 0,0264797 |
| Sb04g008670 | MYB-like HTH transcriptional regulator | 1,44684 | 0 | #NOME? | 0,0264797 |
| Sb04g009670 | BAG6 (BCL-2-associated athanogene 6) | 6,80622 | 27,3091 | 2,00446 | 0,0264797 |
| Sb04g009690 | BAG5 (BCL-2-associated athanogene 5) | 129,521 | 406,876 | 1,65141 | 0,0264797 |
| Sb04g001130 | CAT1 - Catalase isozyme A | 57,9455 | 14,5686 | -1,99184 | 0,0477927 |
| Sb04g026430 | RNA-binding (RRM/RBD/RNP motifs) family protein | 57,6438 | 183,417 | 1,66989 | 0,0264797 |
| Sb04g027330 | HSP20-like - Heat shock | 96,6096 | 669,184 | 2,79217 | 0,0264797 |
| Sb05g000440 | ASN1 - Asparagine synthetase – (DIN6) | 108,814 | 32,1247 | -1,76011 | 0,0264797 |
| Sb05g004100 | ASR1 - Abscisic stress-ripening 1 | 288,25 | 60,0521 | -2,26303 | 0,0264797 |
| Sb06g024780 | LTPL120 - Protease inhibitor/seed storage/LTP family protein | 0 | 8,15305 | inf | 0,0264797 |
| Sb06g024790 | LTPL120 - Protease inhibitor/seed storage/LTP family protein | 10,1617 | 485,377 | 5,57789 | 0,0264797 |
| Sb06g032310 | Leucine-rich repeat (LRR) family protein | 84,8772 | 27,8199 | -1,60926 | 0,0264797 |

“NI” means no identified; “inf” indicates no ratio.

**Table S2.** Continuation.

| **GeneID** | **Annotation** | **ScGAIOE** | **HpScGAI** | **Fold change (Log2)** | **q-values** |
| --- | --- | --- | --- | --- | --- |
| Sb06g033030 | PORA - Protochlorophyllide reductase A | 329,404 | 100,367 | -1,71457 | 0,0264797 |
| Sb06g000660 | HSP83 - Heat shock protein | 24,7329 | 521,226 | 4,39741 | 0,0264797 |
| Sb07g020270 | TPS9 - Trehalose-6-phosphatase synthase 9 | 15,7126 | 3,433 | -2,19438 | 0,0264797 |
| Sb09g023060 | PDC1 – Pyruvate decarboxylase 1 | 7,68714 | 39,1058 | 2,34686 | 0,0264797 |
| Sb09g019930 | PPDK - Pyruvate orthophosphate dikinase | 6,44379 | 25,8605 | 2,00477 | 0,0264797 |
| Sb09g022260 | Putative uncharacterized protein | 58,5064 | 8,82706 | -2,72859 | 0,0264797 |
| Sb09g025900 | HSP101 - Heat shock protein | 87,2806 | 457,537 | 2,39016 | 0,0264797 |
| Sb09g029500 | Pectin lyase-like superfamily protein | 26,2983 | 111,757 | 2,08733 | 0,0264797 |

“NI” means no identified; “inf” indicates no ratio.

**Table S3.** Composite list of DEGs in internode 5^th^ between ScGAIOE and HpScGAI.

| **GeneID** | **Annotation** | **ScGAIOE** | **HpScGAI** | **Fold change (Log2)** | **q-values** |
| --- | --- | --- | --- | --- | --- |
| Sb01g009080 | GRF1-interacting factor 1 | 0 | 1,76847 | inf | 0,035812 |
| Sb01g020430 | Glycine-rich protein DOT1 | 110,538 | 449,668 | 2,02431 | 0,022577 |
| Sb01g036020 | CBL-interacting serine/threonine-protein kinase 10 – CIPK10 | 67,7524 | 15,3461 | -2,1424 | 0,022577 |
| Sb01g043630 | Dwarf 14 - D14 | 131,511 | 28,1199 | -2,22552 | 0,022577 |
| Sb01g047020 | Calmodulin-binding receptor-like cytoplasmic kinase 2 –CRCK2 | 29,0656 | 5,49055 | -2,40429 | 0,035812 |
| Sb01g047160 | RZFP34/CHYR1 | 113,677 | 27,805 | -2,03152 | 0,035812 |
| - | NI | 0 | 4,80613 | inf | 0,022577 |
| - | NI | 517,286 | 2730,2 | 2,39997 | 0,022577 |
| Sb10g012970 | Peptidyl-prolyl cis-trans isomerase FKBP65 – ROF2 | 42,3328 | 999,134 | 4,56083 | 0,022577 |
| Sb10g023670 | EIN3-binding F-box protein 1 – EBF1 | 132,585 | 30,6841 | -2,11135 | 0,022577 |
| Sb10g026090 | Chloride channel protein CLC-a | 37,4577 | 3,88015 | -3,27108 | 0,022577 |
| Sb02g033240 | xyloglucan endotransglucosylase/hydrolase protein 32 – XTH32 | 30,1725 | 107,884 | 1,83818 | 0,035812 |
| Sb02g003010 | Early-responsive to dehydration 4 - ERD4 | 90,6823 | 20,9488 | -2,11396 | 0,022577 |
| Sb02g031550 | Copper amine oxidase family protein | 0 | 4,77988 | inf | 0,022577 |
| - | NI | 0 | 53,1402 | inf | 0,022577 |
| Sb03g042860 | AKS2- ABA-responsive kinase substrate 2 | 73,8751 | 13,9807 | -2,40166 | 0,022577 |
| Sb03g002020 | NA^+^/CA^2+^ Exchanger | 268,712 | 75,106 | -1,83906 | 0,035812 |
| Sb03g039530 | Laccase-17 – LAC17 | 31,3293 | 3,59022 | -3,12537 | 0,022577 |
| Sb04g009670 | BAG family molecular chaperone regulator 6 – BAG6 | 8,12306 | 31,1694 | 1,94004 | 0,035812 |
| Sb04g017450 | Inositol-tetrakisphosphate 1-kinase 1 – ITPK1 | 0 | 1,75114 | inf | 0,035812 |
| Sb04g024090 | Protein NRT1/ PTR FAMILY 6.4 –NPF6.4 | 45,4655 | 4,44484 | -3,35457 | 0,022577 |
| Sb04g035560 | TPS11 – Trehalose-6-phosphatase synthase 11 | 76,4698 | 20,5569 | -1,89527 | 0,022577 |
| Sb04g021590 | Copper transport protein- CCH | 23,4383 | 170,208 | 2,86036 | 0,022577 |
| Sb04g027330 | heat shock protein- HSP23.5 | 52,6493 | 545,481 | 3,37304 | 0,022577 |
| - | NI | 0 | 21,772 | inf | 0,022577 |
| - | NI | 0 | 32,9927 | inf | 0,022577 |
| Sb05g007030 | heat shock protein –HSP22 | 0 | 5,28697 | inf | 0,022577 |
| Sb05g008440 | carboxylesterase 17 –CXE17 | 3,2307 | 0 | #NOME? | 0,022577 |
| - | NI | 0 | 5,99304 | inf | 0,022577 |
| - | NI | 0 | 5,65168 | inf | 0,022577 |
| - | NI | 0 | 4,18415 | inf | 0,035812 |
| Sb06g001410 | Pectate lyase 15 | 17,9248 | 83,0608 | 2,21221 | 0,022577 |
| Sb06g002500 | Hypothetical protein | 117,531 | 1082,81 | 3,20367 | 0,022577 |

“NI” means no identified; “inf” indicates no ratio.

**Table S3.** Continuation

| **GeneID** | **Annotation** | **ScGAIOE** | **HpScGAI** | **Fold change (Log2)** | **q-values** |
| --- | --- | --- | --- | --- | --- |
| Sb06g021870 | Leucine-rich repeat (LRR) family protein | 11,792 | 52,4498 | 2,15313 | 0,022577 |
| Sb06g024770 | Bifunctional inhibitor/lipid-transfer protein/seed storage 2S albumin superfamily protein –LTPL121 | 0 | 175,476 | inf | 0,022577 |
| Sb06g024780 | Bifunctional inhibitor/lipid-transfer protein/seed storage 2S albumin superfamily protein –LTPL120 | 0 | 16,2181 | inf | 0,022577 |
| Sb06g025890 | Ethylene-responsive transcription factor ERF025 (DREB A-4) | 7,01096 | 0 | inf | 0,022577 |
| Sb06g028200 | Bifunctional inhibitor/lipid-transfer protein/seed storage 2S albumin superfamily protein | 90,7623 | 463,277 | 2,35171 | 0,022577 |
| Sb06g000660 | Heat shock protein 90-1 – HSP90.1 | 32,6025 | 393,163 | 3,59207 | 0,022577 |
| - | NI | 35,2548 | 274,777 | 2,96237 | 0,022577 |
| - | NI | 450,491 | 3722,99 | 3,04689 | 0,022577 |
| - | NI | 224,836 | 1937,85 | 3,10751 | 0,022577 |
| - | NI | 215,998 | 1309,36 | 2,59977 | 0,035812 |
| - | NI | 144,461 | 1120,93 | 2,95594 | 0,022577 |
| - | NI | 0 | 107,561 | inf | 0,022577 |
| Sb07g020270 | TPS9 - Trehalose-6-phosphatase synthase 9 | 42,6613 | 3,28255 | -3,70004 | 0,022577 |
| Sb08g000980 | Peroxidase 52- PRX52 | 127,791 | 11,9137 | -3,42309 | 0,035812 |
| Sb08g002740 | CBL-interacting serine/threonine-protein kinase 2 – CIPK2 | 95,0556 | 26,1821 | -1,86019 | 0,035812 |
| Sb08g020600 | BZIP63- Basic leucine zipper 63 | 54,0344 | 9,3616 | -2,52905 | 0,022577 |
| Sb08g021580 | APG1 – Albino or pale green mutant 1 | 47,5267 | 6,52539 | -2,8646 | 0,022577 |
| Sb09g023060 | Pyruvate decarboxylase 1 –PDC1 | 1,95158 | 28,5127 | 3,86889 | 0,035812 |
| Sb09g029610 | ADPGLC-PPASE large subunit - APL2 | 44,3375 | 158,499 | 1,83788 | 0,022577 |
| Sb09g018080 | Transducin/WD-40 repeat-containing protein | 10,8563 | 40,7806 | 1,90935 | 0,022577 |
| Sb09g022260 | Unknown protein | 428 | 43,4989 | -3,29856 | 0,022577 |
| Sb09g024060 | KINβ1 - SNF1-related protein kinase regulatory subunit beta-1 | 226,435 | 38,5695 | -2,55356 | 0,022577 |
| Sb09g024230 | CYS6 - Cysteine proteinase inhibitor 6 | 63,9594 | 240,365 | 1,91 | 0,022577 |
| Sb09g029500 | PG2 - Polygalacturonase | 7,38358 | 47,9777 | 2,69997 | 0,022577 |
| - | NI | 0 | 76,565 | inf | 0,022577 |

“NI” means no identified; “inf” indicates no ratio.

**Table S4.** Composite list of DEGs in internode 9^th^ between ScGAIOE and HpScGAI.

| **GeneID** | **Annotation** | **ScGAIOE** | **HpScGAI** | **Fold change (Log2)** | **q-values** |
| --- | --- | --- | --- | --- | --- |
| Sb01g000580 | GA20ox2 - Gibberellin 20 oxidase 2 | 1,48898 | 0 | inf | 0,009611 |
| Sb01g004295 | SMT1 – Sterol methyltransferase 1 | 61,391 | 0 | inf | 0,023238 |
| Sb01g007170 | ZAT6 - Zinc finger protein | 4,21876 | 75,7109 | 4,16561 | 0,023238 |
| Sb01g007340 | PP2C clade D5 – ADP5 | 30,8246 | 241,756 | 2,9714 | 0,009611 |
| Sb01g013190 | SPX (SYG1/Pho81/XPR1) domain-containing protein / zinc finger (C3HC4-type RING finger) protein-related | 5,91843 | 0 | inf | 0,009611 |
| Sb01g014350 | Cytochrome P450, family 87, subfamily A, polypeptide 2 – CYP87A2 | 12,3886 | 57,9573 | 2,22598 | 0,033482 |
| Sb01g020430 | Unknown protein | 129,822 | 16,4984 | -2,97613 | 0,009611 |
| Sb01g020570 | PHT1.4 -Inorganic phosphate transporter 1-4 | 16,15 | 82,9606 | 2,36089 | 0,023238 |
| Sb01g021130 | GDSL esterase/lipase EXL3 | 3,00806 | 0 | inf | 0,009611 |
| Sb01g027285 | PSBC - Photosystem II reaction center protein C | 0 | 15,7927 | inf | 0,033482 |
| Sb01g027810 | Early-responsive to dehydration – (ERD4) | 38,451 | 8,18857 | -2,23134 | 0,033482 |
| Sb01g029330 | EXPβ2 - Expansin-β2 | 3,53089 | 0 | inf | 0,017156 |
| Sb01g032610 | Terpene synthase –TPS21 | 15,0758 | 114,789 | 2,92867 | 0,009611 |
| Sb01g035970 | Putative uncharacterized protein | 2,17049 | 0 | inf | 0,037292 |
| Sb01g043030 | BGLU40 - Beta-glucosidase 40 | 1,65775 | 0 | inf | 0,017156 |
| Sb01g001160 | CYP71B23 - Cytochrome P450 | 1,13987 | 0 | inf | 0,023238 |
| Sb01g003270 | C2H2-type zinc finger family protein | 0 | 8,10497 | inf | 0,009611 |
| Sb01g003280 | C2H2-type zinc finger family protein | 0 | 8,15342 | inf | 0,009611 |
| Sb01g003710 | ATAF2 – NAC domain protein | 12,1639 | 161,214 | 3,7283 | 0,009611 |
| Sb01g004320 | Cupredoxin superfamily protein | 3,59622 | 0 | inf | 0,009611 |
| Sb01g004740 | AAA-ATPASE 1 | 25,9359 | 194,583 | 2,90736 | 0,009611 |
| Sb01g005900 | Syntaxin-121 | 11,6421 | 84,0838 | 2,85247 | 0,009611 |
| Sb01g007220 | Putative uncharacterized protein | 0 | 83,1714 | inf | 0,009611 |
| Sb01g008350 | BIL4 - BRZ-Insensitive-long hypocotyls 4 | 29,4707 | 285,007 | 3,27364 | 0,009611 |
| Sb01g010050 | Uncharacterized protein | 33,4779 | 220,235 | 2,71776 | 0,009611 |
| Sb01g013270 | YAB1 - Axial regulator YABBY 1 | 5,88927 | 0 | inf | 0,045876 |
| Sb01g014120 | SHY2/IAA3 - Auxin-responsive protein | 1,9053 | 0 | inf | 0,037292 |
| Sb01g015070 | Terpenoid cyclases | 0,980916 | 0 | inf | 0,037292 |
| Sb01g018360 | ABCG11 - ABC transporter G family member 11 | 1,18957 | 0 | inf | 0,017156 |
| Sb01g036020 | CBL-interacting serine/threonine-protein kinase 10 – CIPK10 | 53,221 | 10,0121 | -2,41025 | 0,029034 |
| Sb01g037090 | GOLS1 - Galactinol synthase 1 | 14,5121 | 85,3798 | 2,55664 | 0,009611 |
| Sb01g037850 | COBL7 - COBRA-like protein 7 | 5,25721 | 50,5362 | 3,26495 | 0,009611 |
| Sb01g038410 | AP2C1 - PP2C clade B | 18,8677 | 500,749 | 4,7301 | 0,009611 |

“inf” indicates no ratio.

**Table S4.** Continuation.

| **GeneID** | **Annotation** | **ScGAIOE** | **HpScGAI** | **Fold change (Log2)** | **q-values** |
| --- | --- | --- | --- | --- | --- |
| Sb01g039530 | HSP70 - Heat shock protein | 74,932 | 394,979 | 2,39812 | 0,009611 |
| Sb01g041310 | Glycosyl hydrolase family 10 | 1,31809 | 0 | inf | 0,009611 |
| Sb01g043460 | Uncharacterized protein | 57,5594 | 228,242 | 1,98744 | 0,029034 |
| - | NI | 29,0104 | 161,221 | 2,4744 | 0,009611 |
| Sb10g000980 | CSLD3 - Cellulose synthase-like protein D3 | 13,2009 | 76,3735 | 2,53243 | 0,009611 |
| Sb10g001620 | CBF3 - Dehydration-responsive element-binding protein 1A | 0 | 1,86575 | inf | 0,041518 |
| Sb10g003340 | GDSL esterase/lipase | 1,00144 | 0 | inf | 0,045876 |
| Sb10g003890 | GDSL esterase/lipase | 265,057 | 47,8687 | -2,46915 | 0,009611 |
| Sb10g012970 | Peptidyl-prolyl cis-trans isomerase FKBP65 – ROF2 | 23,5841 | 706,669 | 4,90515 | 0,009611 |
| Sb10g023970 | Uncharacterized protein | 86,9415 | 10,8877 | -2,99735 | 0,009611 |
| Sb10g025210 | LTPL129 - Protease inhibitor/seed storage/LTP family protein | 1,70765 | 0 | inf | 0,009611 |
| Sb10g027000 | Patellin-4 | 1,42238 | 0 | inf | 0,009611 |
| Sb10g000660 | Pectate lyase-like superfamily protein | 83,7454 | 16,5356 | -2,34043 | 0,009611 |
| Sb10g002070 | ADC2 - Arginine decarboxylase 2 | 8,75275 | 46,4398 | 2,40755 | 0,029034 |
| Sb10g004240 | UDGT71C5 - UDP-glycosyltransferase 71C5 | 1,09525 | 0 | inf | 0,037292 |
| Sb10g006630 | Putative uncharacterized | 6,03264 | 73,3782 | 3,60449 | 0,009611 |
| Sb10g008130 | FTSH6 - ATP-dependent zinc metalloprotease | 5,40909 | 33,3836 | 2,62568 | 0,029034 |
| Sb10g012220 | BGLU17 - Beta-glucosidase 17 | 50,3167 | 4,58139 | -3,45718 | 0,017156 |
| - | NI | 35,7038 | 0 | inf | 0,009611 |
| Sb02g009340 | Putative lipid-transfer protein DIR1 | 0 | 32,7424 | inf | 0,023238 |
| Sb02g022290 | WRKY53 | 3,48877 | 80,0604 | 4,5203 | 0,009611 |
| Sb02g028240 | SAP5 – Stress associated protein 5 | 23,4327 | 126,628 | 2,434 | 0,009611 |
| Sb02g033240 | xyloglucan endotransglucosylase/hydrolase protein 32 – XTH32 | 5,67153 | 0 | inf | 0,009611 |
| Sb02g035460 | O-Glycosyl hydrolases family 17 protein | 2,16182 | 0 | inf | 0,009611 |
| Sb02g001740 | Uncharacterized protein | 1,2766 | 0 | inf | 0,017156 |
| Sb02g004390 | ELIP1 - Early light-induced protein 1, chloroplastic | 0 | 6,35111 | inf | 0,009611 |
| Sb02g004670 | SHY2/IAA3 - Auxin-responsive protein | 20,1516 | 248,64 | 3,62509 | 0,009611 |
| Sb02g005780 | GALT6 – O-galactosyltransferase | 3,79149 | 29,0339 | 2,9369 | 0,033482 |
| Sb02g009600 | CRSP – CO2-response secreted protease | 49,9568 | 3,84074 | -3,70122 | 0,009611 |
| Sb02g023660 | Glycosyl hydrolase family 81 protein | 5,3742 | 38,8393 | 2,85339 | 0,009611 |
| Sb02g023910 | SAP12 – Stress associated protein 12 | 0 | 7,34847 | inf | 0,009611 |
| Sb02g031550 | Copper amine oxidase family protein | 0 | 6,2105 | inf | 0,009611 |
| Sb02g035930 | B-120 -G-type lectin S-receptor-like serine/threonine-protein kinase | 0 | 1,01966 | inf | 0,009611 |

“NI” means no identified; “inf” indicates no ratio.

**Table S4.** Continuation.

| **GeneID** | **Annotation** | **ScGAIOE** | **HpScGAI** | **Fold change (Log2)** | **q-values** |
| --- | --- | --- | --- | --- | --- |
| Sb02g036605 | Unknown protein | 0 | 7,34847 | inf | 0,009611 |
| Sb02g037350 | OSM34 -Osmotin-like protein | 1,52028 | 0 | inf | 0,033482 |
| Sb02g042280 | GDSL esterase/lipase | 2,92582 | 0 | inf | 0,009611 |
| Sb02g043060 | Putative uncharacterized protein | 2,41391 | 116,664 | 5,59484 | 0,009611 |
| - | NI | 0 | 68,2309 | inf | 0,023238 |
| Sb03g003000 | Unknown protein | 7,38097 | 43,0973 | 2,54571 | 0,037292 |
| Sb03g003530 | HSP17.6II - Heat shock | 18,5509 | 136,753 | 2,88201 | 0,009611 |
| Sb03g006880 | HSP18.2 - Heat shock | 149,763 | 1297,54 | 3,11503 | 0,009611 |
| Sb03g013210 | Peroxidase superfamily protein | 1,44158 | 0 | inf | 0,037292 |
| Sb03g026050 | Unknown protein | 2,10569 | 56,9051 | 4,75619 | 0,017156 |
| Sb03g029790 | EMB2742 – Embryo defective 2742 | 19,6885 | 1,34664 | -3,86992 | 0,023238 |
| Sb03g030340 | MAN7 - Mannan endo-1,4-beta-mannosidase 7 | 1,94234 | 0 | inf | 0,009611 |
| Sb03g037080 | ERF9 - Ethylene-responsive transcription factor 9 | 23,8588 | 173,638 | 2,86349 | 0,009611 |
| Sb03g038290 | EXPA8 – Expansin A8 | 9,11544 | 0 | inf | 0,009611 |
| Sb03g038880 | 2-oxoglutarate (2OG) and Fe(II)-dependent oxygenase superfamily protein | 1,81198 | 0 | inf | 0,009611 |
| Sb03g041100 | CP22 Photosystem II subunit S | 16,5613 | 131,253 | 2,98646 | 0,009611 |
| Sb03g003310 | Unknown protein | 3,70572 | 186,675 | 5,65463 | 0,009611 |
| Sb03g006870 | HSP18.2 - Heat shock | 254,858 | 1830,2 | 2,84424 | 0,009611 |
| Sb03g020184 | GRP1 – Glycine-rich RNA binding protein 1 | 5,2335 | 44,5266 | 3,08882 | 0,017156 |
| Sb03g025730 | Calmodulin-binding family protein | 1,92449 | 62,3619 | 5,01812 | 0,029034 |
| Sb03g029520 | AKT1 – K^+^ transporter | 1,90883 | 18,1015 | 3,24535 | 0,023238 |
| Sb03g032140 | MAPKKK17 - Mitogen-activated protein kinase kinase kinase 17 | 0 | 3,77935 | inf | 0,009611 |
| Sb03g034090 | OXS3 – Oxidative stress 3 | 57,4861 | 298,396 | 2,37594 | 0,009611 |
| Sb03g039330 | Pathogenesis-related thaumatin superfamily protein | 1,73687 | 0 | inf | 0,041518 |
| Sb03g040300 | HSPRO1 - Nematode resistance protein-like | 4,23374 | 88,2553 | 4,38168 | 0,017156 |
| Sb03g040950 | ASFT- Aliphatic suberin feruloyl-transferase | 2,04455 | 0 | inf | 0,009611 |
| Sb03g043430 | Unknown protein | 321,263 | 1793,6 | 2,48103 | 0,009611 |
| Sb03g045000 | Calcium-dependent lipid-binding (CaLB domain) family protein | 3,19283 | 57,1091 | 4,16081 | 0,041518 |
| - | NI | 0 | 50,8151 | inf | 0,041518 |
| - | NI | 0 | 16,6245 | inf | 0,009611 |
| Sb04g002950 | SRF1 - Strubbelig-receptor family 1 | 0,949692 | 0 | inf | 0,033482 |
| Sb04g005520 | WRKY40 | 7,67975 | 66,5328 | 3,11493 | 0,009611 |
| Sb04g008670 | Myb-like HTH transcriptional regulator-like protein | 1,26062 | 0 | inf | 0,017156 |

“NI” means no identified; “inf” indicates no ratio.

**Table S4.** Continuation.

| **GenID** | **Annotation** | **ScGAIOE** | **HpScGAI** | **Fold change (Log2)** | **q-values** |
| --- | --- | --- | --- | --- | --- |
| Sb04g009690 | UGT84B2 - UDP-glucosyl transferase 84B2 | 272,006 | 1080,61 | 1,99014 | 0,045876 |
| Sb04g026560 | PAL1 - Phenylalanine ammonia-lyase 1 | 2,70701 | 32,8481 | 3,60104 | 0,033482 |
| Sb04g028460 | GATL9 - Galacturonosyltransferase-like 9 | 7,07327 | 117,151 | 4,04985 | 0,009611 |
| Sb04g028490 | Actin-binding FH2 (formin homology 2) family protein | 5,46972 | 47,7885 | 3,12713 | 0,033482 |
| Sb04g028830 | SGNH hydrolase-type esterase superfamily protein | 5,40017 | 0 | inf | 0,009611 |
| Sb04g030080 | GALS1 – Galactan synthase 1 | 29,9729 | 162,196 | 2,43601 | 0,029034 |
| Sb04g032250 | ERD10 – Early responsive to dehydration 10 | 43,0117 | 201,862 | 2,23057 | 0,023238 |
| Sb04g032820 | EXPβ4 - Expansin-β4 | 1,83753 | 0 | inf | 0,009611 |
| Sb04g035630 | GAE1 - UDP-glucuronate 4-epimerase 1 | 6,30856 | 50,7312 | 3,00749 | 0,009611 |
| Sb04g035810 | BRS1 - BRI1 suppressor 1 | 2,83118 | 0 | inf | 0,009611 |
| Sb04g007190 | KCS11 - 3-ketoacyl-CoA synthase 11 | 4,05567 | 41,0833 | 3,34054 | 0,009611 |
| Sb04g007230 | UGT73C6 - UDP-glycosyltransferase 73C6 | 1,03048 | 0 | inf | 0,041518 |
| Sb04g008110 | Leucine-rich repeat receptor-like protein kinase | 36,2994 | 3,48495 | -3,38073 | 0,009611 |
| Sb04g015420 | SWEET7 - Bidirectional sugar transporter | 212,628 | 50,4851 | -2,0744 | 0,037292 |
| Sb04g027330 | HSP20 - Heat shock protein | 21,7175 | 429,757 | 4,30659 | 0,009611 |
| Sb04g029960 | XCP1 - Xylem cysteine proteinase 1 | 14,063 | 0 | inf | 0,009611 |
| Sb04g032830 | EXPβ4 - Expansin-β4 | 9,63691 | 0 | inf | 0,009611 |
| Sb04g033150 | DALL1 - Phospholipase A1-Ibeta2 | 5,62398 | 65,7296 | 3,54688 | 0,009611 |
| Sb04g036920 | PPPDE putative thiol peptidase family protein | 39,4882 | 297,702 | 2,91438 | 0,009611 |
| - | NI | 0 | 28,1379 | inf | 0,009611 |
| Sb05g002640 | Ankyrin repeat-containing protein | 3,95258 | 82,5697 | 4,38475 | 0,009611 |
| Sb05g017960 | HSD1 - 11-beta-hydroxysteroid dehydrogenase 1B | 2,19215 | 0 | inf | 0,009611 |
| Sb05g019180 | TPS21 - Terpene synthase | 0 | 4,2059 | inf | 0,009611 |
| Sb05g022580 | Subtilisin-like protease | 2,5171 | 0 | inf | 0,009611 |
| Sb05g022620 | Subtilisin-like protease | 90,7985 | 9,38795 | -3,27379 | 0,009611 |
| - | NI | 0 | 6,62481 | inf | 0,009611 |
| - | NI | 0 | 12,8641 | inf | 0,009611 |
| - | NI | 0 | 20,9474 | inf | 0,029034 |
| Sb06g001970 | APX3- Ascorbate peroxidase 3 | 0 | 8,9377 | inf | 0,009611 |
| Sb06g002500 | Unknown protein | 373,849 | 0 | inf | 0,009611 |
| Sb06g024110 | Homeodomain-like superfamily protein | 16,6101 | 175,367 | 3,40025 | 0,009611 |
| Sb06g025870 | MATE efflux family protein | 4,61271 | 41,5433 | 3,17093 | 0,029034 |
| Sb06g028090 | ERF7 - Ethylene-responsive transcription factor 7 | 6,68328 | 101,31 | 3,92208 | 0,009611 |

“NI” means no identified; “inf” indicates no ratio.

**Table S4.** Continuation.

| **GeneID** | **Annotation** | **ScGAIOE** | **HpScGAI** | **Fold change (Log2)** | **q-values** |
| --- | --- | --- | --- | --- | --- |
| Sb06g033520 | CCR4-associated factor 1B | 6,62306 | 120,969 | 4,191 | 0,009611 |
| Sb06g000660 | HSP90.1 - Heat shock | 19,1757 | 356,74 | 4,21752 | 0,009611 |
| Sb06g015940 | XTH25 - Xyloglucan endotransglucosylase/hydrolase protein 25 | 15,8663 | 106,42 | 2,74573 | 0,009611 |
| Sb06g022880 | GA2OX8 - Gibberellin 2-beta-dioxygenase 8 | 0 | 2,33684 | inf | 0,017156 |
| Sb06g025170 | CYP86A4 - Cytochrome P450 86A4 | 1,06864 | 0 | inf | 0,023238 |
| Sb06g026160 | ACS6 - 1-aminocyclopropane-1-carboxylate synthase 6 | 2,62987 | 46,1604 | 4,13359 | 0,009611 |
| Sb06g031300 | Peroxidase superfamily protein | 1,4164 | 0 | inf | 0,017156 |
| Sb06g033570 | NDR1/HIN1-LIKE 2 | 59,3943 | 275,639 | 2,21438 | 0,023238 |
| - | NI | 164,003 | 0 | inf | 0,037292 |
| - | NI | 64,7768 | 0 | inf | 0,009611 |
| - | NI | 1037,36 | 0 | inf | 0,009611 |
| - | NI | 532,815 | 0 | inf | 0,009611 |
| - | NI | 173,809 | 0 | inf | 0,009611 |
| - | NI | 325,53 | 0 | inf | 0,009611 |
| - | NI | 421,92 | 0 | inf | 0,009611 |
| - | NI | 18,1711 | 0 | inf | 0,037292 |
| Sb07g000510 | CYP71B34 - Cytochrome P450 71B34 | 1,92836 | 0 | inf | 0,017156 |
| Sb07g023030 | ERF109 - Ethylene-responsive transcription factor109 | 0 | 5,07173 | inf | 0,023238 |
| Sb07g001090 | Core-2/I-branching beta-1,6-N-acetylglucosaminyltransferase family protein | 18,7012 | 231,186 | 3,62785 | 0,009611 |
| Sb07g009580 | Eukaryotic aspartyl protease family protein | 0,835311 | 0 | inf | 0,045876 |
| Sb07g020270 | TPS9 - Trehalose-6-phosphatase synthase 9 | 31,7242 | 5,11352 | -2,63319 | 0,045876 |
| Sb07g023210 | PLP2 - Patatin-like protein 2 | 0 | 0,903477 | inf | 0,041518 |
| Sb07g023340 | UCP5 - Mitochondrial uncoupling protein 5 | 32,0565 | 198,451 | 2,6301 | 0,009611 |
| Sb08g004980 | AAP3 - Amino acid permease 3 | 6,62457 | 49,2703 | 2,89482 | 0,017156 |
| Sb08g005680 | HXXXD-type acyl-transferase family protein | 1,39852 | 0 | inf | 0,037292 |
| Sb08g015237 | CRK10 - Cysteine-rich receptor-like protein kinase 10 | 1,52622 | 0 | inf | 0,033482 |
| Sb08g004960 | AAP3 - Amino acid permease 3 | 9,06995 | 79,2295 | 3,12687 | 0,009611 |
| Sb08g021800 | LATE FLOWERING | 6,28197 | 0 | inf | 0,009611 |
| Sb08g022450 | OSM34 – Osmotin 34 | 1,56038 | 0 | inf | 0,041518 |
| - | NI | 0 | 26,0123 | inf | 0,009611 |
| - | NI | 0 | 16,652 | inf | 0,041518 |
| Sb09g002390 | TZF9 – Tandem Zinc finger protein 9 | 19,2684 | 164,072 | 3,09002 | 0,009611 |
| Sb09g003230 | Phosphoglycerate mutase family protein | 41,2426 | 197,18 | 2,25731 | 0,033482 |

“NI” means no identified; “inf” indicates no ratio.

**Table S4.** Continuation.

| **GeneID** | **Annotation** | **ScGAIOE** | **HpScGAI** | **Fold change (Log2)** | **q-values** |
| --- | --- | --- | --- | --- | --- |
| Sb09g006030 | PFK3 - Phosphofructokinase 3 | 0 | 7,30325 | inf | 0,009611 |
| Sb09g015900 | WRKY33 | 5,95004 | 64,2507 | 3,43274 | 0,009611 |
| Sb09g017190 | Glycine-rich protein family | 8,96748 | 0 | inf | 0,009611 |
| Sb09g023380 | ARM repeat superfamily protein | 1,51129 | 75,3009 | 5,63881 | 0,009611 |
| Sb09g025090 | Protein of unknown function (DUF567) | 0 | 2,78348 | inf | 0,033482 |
| Sb09g026830 | WRKY51 | 5,3416 | 49,6088 | 3,21525 | 0,009611 |
| Sb09g000270 | CYP722A1 - Cytochrome P450, family 722, subfamily A, polypeptide 1 | 33,9448 | 5,2331 | -2,69745 | 0,045876 |
| Sb09g003060 | RCI2A - Rare-cold-inducible 2a | 99,5521 | 501,7 | 2,3333 | 0,037292 |
| Sb09g005340 | Unknown protein | 0 | 16,4329 | inf | 0,009611 |
| Sb09g019930 | PPDK - Pyruvate orthophosphate dikinase 1 | 29,8375 | 129,252 | 2,11498 | 0,023238 |
| Sb09g022260 | Unknown protein | 765,238 | 75,2771 | -3,34562 | 0,009611 |
| Sb09g027360 | PMEAMT - Phosphomethylethanolamine N-methyltransferase | 98,5499 | 6,18125 | -3,99488 | 0,009611 |
| Sb09g029130 | CTP synthase | 56,7059 | 14,5123 | -1,96622 | 0,037292 |
| Sb09g029575 | RL6 - Protein RADIALIS-like 6 | 24,0965 | 0 | inf | 0,033482 |
| Sb09g029860 | LEA27 – Late embryogenesis abundant 27 | 85,0088 | 516,241 | 2,60236 | 0,009611 |
| - | NI | 0 | 62,1164 | inf | 0,029034 |

“NI” means no identified; “inf” indicates no ratio.

**Table S5.** Primers used in this study.

| **Primer** | **Sequences** | **Used for** |
| --- | --- | --- |
|  |  |  |
| ScUbi-1_fw | 5’-CCGGTCCTTTAAACCAACTCAGT-3’ | cDNA |
| ScUbi-1_rev | 5’-CCCTCTGGTGTACCTCCATTTG-3’ |  |
| ScGAI_fw | 5’-CATATGAAGCGCGAGTACCAAGACGC-3’ | Cloning and YTH |
| ScGAI_rev | 5’-CTGCAGCCCCACCCCTCGATCAC-3’ |  |
| ScPIF3_fw | 5’- CATATGTCCGACGGCAACGAGT -3’ | Cloning and YTH |
| ScPIF3_rev | 5’-CTCGAGGCTGACTGTTTTTATGTTTCAGCT-3’ |  |
| ScPIF4_fw | 5’- CATatggaCGGCAATgcgag-3’ | Cloning and YTH |
| ScPIF4_rev | 5’-GAGCTCTTACGAGATTTTCCTCATTCTAAAC-3’ |  |
| ScPIF5_fw | 5’-CgcccCATatgaaccaG-3’ | Cloning and YTH |
| ScPIF5_rev | 5’- GGATCCATCAACCTAACACCATCATATCA-3’ |  |
| ScGAI_fw | 5’-TCTAGAATGAAGCGCGAGTACCAAGACGC-3’ | BIFC |
| ScGAI_rev | 5’-CCCGGGCCCACCCCTCGACGGAGC-3’ |  |
| ScGAI_trunc_fw | 5’-TCTAGAATGCGCAAGGTCGCCGCCTACTT-3’ | BIFC |
| ScPIF3_fw | 5’- TCTAGAATGTCCGACGGCAACGAGT -3’ |  |
| ScPIF3_rev | 5’-GTCGACTGTTTCAGCTTCATTTCTTCC-3’ | BIFC |
| ScPIF4_fw | 5’- TCTAGAatggaCGGCAATgcgag-3’ |  |
| ScPIF4_rev | 5’-GTCGACAACTCCAAAAGTAGGTGG-3’ | BIFC |
| oxScGAI_fw | 5’-GATATCGTAAACCATGGACTACAAGGACGACGATGACAAAATGAAGCGCGAGTACCAAGACGC-3’ | ScGAI overexpressing |
| oxScGAI_rev | 5’-GGGGTACCCCCCACCCCTCGATCAC-3’ |  |
| asScGAI_fw | 5’-CGGGATCCGGATGACGACGAGGAAGAGGAA-3’ | ScGAI silencing |
| asScGAI_rev | 5’-GGCCAGATATCGAGGAGATGGACGAGATGCT-3’ |  |
| sScGAI_fw | 5’-CGACGCGTCGAGGAGATGGACGAGATGCT-3’ | ScGAI silencing |
| sScGAI_rev | 5’-GGGGTACCCCGGATGACGACGAGGAAGAGGAA-3’ |  |
| IntronII_fw | 5’-GGCCAGATATCATGCGGTAACTGATCTGAATT-3’ | ScGAI silencing |
| IntronII_rev | 5’-CGACGCGTCACCTGCAGAGTGTGTAGATAA-3’ |  |
| ScEIN3_fw | 5’-AAAATCTAGAATGATGGGAGGCGGGCTGATGA-3’ | Cloning and BIFC |
| ScEIN3_rev | 5’-AAAACCCGGGGTAGAACCAATTGGTCCCGTCGT-3’ |  |
| ScEIL1_fw | 5’-AAAATCTAGATACCTCTACGCTCGGCGTGATG-3’ | Cloning and BIFC |
| ScEIL1_rev | 5’-AAAACCCGGGATTCTGCCGCAGGTAGAACCAATT-3’ |  |
| ScEIN3_fw | 5’-AAAACATATGATGGGAGGCGGGCTGATGA -3’ | YTH |
| ScEIN3_rev | 5’-AAAACTCGAGTCAGTAGAACCAATTGGTCCCGT -3’ |  |
| ScEIL1_fw | 5’-AAAACATATGATGGGAGGAAGAGGGGC -3’ | YTH |
| ScEIL1_rev | 5’-AAAAGAATTCTCAGTAGAACCAATTGGCGTTGGAT -3’ |  |

**Table S5.** Continuation.

| **Primer** | **Sequences** | **Used for** |
| --- | --- | --- |
| ScEIN3_tr_fw | 5’-CATATGATGCAGCACTGTGACCCCCCACAG -3’ | YTH |
| ScEIN3_tr_rev | 5’-GAATTCTCActggggcatggcattaggcctctc -3’ |  |
| ScGAI_SAW_fw | 5’-GCTGGCACTCTTCAACG-3’ | Arabidopsis screening |
| VENUS_rev | 5’- CCAGCTCGACCAGGATG-3’ |  |
| βactin2_fw | 5’-TTCTCTCCTTGTACGCC -3’ | Arabidopsis screening |
| βactin2_rev | 5’- AACGATTCCTGGACCTGCCTCATC-3’ |  |
| ScPIF3_fw | 5’- GAATTCATGTCCGACGGCAACGAGT -3’ | Subcellular localization |
| ScPIF3_rev | 5’-AAGCTTTGTTTCAGCTTCATTTCTTCC-3’ |  |
| ScPIF4_fw | 5’- AAGCTTATGGACGGCAATGCGAG-3’ | Subcellular localization |
| ScPIF4_rev | 5’-AAGCTTAACTCCAAAAGTAGGTGG-3’ |  |
| Ubi1_intron_fw | 5’-ttgtcgatgctcaccctgttgtttg-3’ | Genotype screening |
| ScGAI_rev | 5’-gggagatcgaagtagccagc-3’ |  |
| ScGAI_fw | 5’- caccgtgcactacaatccct-3’ |  |
| qUTR_della_fw | 5’-CACCTCCGCTTCAAGGTC-3’ | qPCR |
| qUTR_della_rev | 5’-CTTGGTACTCGCGCTTCAT-3’ |  |
| qDELLA_fw | 5’-CCAAGGACAAGATGATGGTG-3’ | qPCR |
| qDELLA_rev | 5’-GACGAACGCACCTTGTACC-3’ |  |
